# APOE4 reshapes the lipid droplet proteome and modulates microglial inflammatory responses

**DOI:** 10.1016/j.nbd.2025.106983

**Published:** 2025-05-30

**Authors:** Cassi M. Friday, Isaiah O. Stephens, Cathryn T. Smith, Sangderk Lee, Diksha Satish, Nicholas A. Devanney, Sarah Cohen, Josh M. Morganti, Scott M. Gordon, Lance A. Johnson

**Affiliations:** a Department of Physiology, University of Kentucky, Lexington, KY, USA; b Sanders Brown Center on Aging, University of Kentucky, Lexington, KY, USA; c Department of Cell Biology & Physiology, University of North Carolina, Chapel Hill, NC, USA; d Department of Neuroscience, University of Kentucky, Lexington, KY, USA; e Saha Cardiovascular Center, University of Kentucky, Lexington, KY, USA

**Keywords:** Apolipoprotein E4, APOE4, Lipid droplets, Microglia, Neuroinflammation, Alzheimer’s disease, Proteomics, Lipidomic, Fatty acid metabolism

## Abstract

Excess lipid droplet (LD) accumulation is implicated in various diseases, including Alzheimer’s disease (AD), yet the mechanisms underlying this accumulation remain unclear. Apolipoprotein E (ApoE) is a droplet-associated protein, and its E4 variant confers the greatest genetic risk for late-onset AD while also being linked to increased neuroinflammation and LD accumulation. In this study, we compared the lipid and protein composition of hepatic LDs in targeted replacement mice expressing human E3 (neutral) or E4 (risk variant), under both baseline conditions and following lipopolysaccharide (LPS) administration. Lipidomic analysis revealed that E4 LDs exhibit a shift in glycerophospholipid distribution, with an increase in phosphatidylcholine species, such that their baseline profile resembles that of LPS-treated LDs. Quantitative proteomics indicated that E4 LDs are enriched in proteins related to vesicle transport but show decreased levels of proteins involved in fatty acid β-oxidation. Notably, many LD-associated proteins overlapped with those identified in AD postmortem and microglial ‘omics studies, suggesting a role for LDs in AD pathogenesis. To further explore these findings, primary microglia from E3 and E4 mice were exposed to exogenous lipids, LPS, and necroptotic N2A cells. Under most conditions, E4 microglia accumulated more LDs and secreted higher levels of proinflammatory cytokines (TNF, IL-1β, IL-10) compared to E3 microglia, although their LPS response was blunted. These data suggest that altered LD dynamics in E4 microglia may contribute to the increased AD risk associated with APOE4.

## 1. Introduction

Lipid droplets (LDs) are ubiquitous storage organelles which are conserved across many species and expressed in multiple cell types (Guo et al., 2009; Olzmann and Carvalho, 2019; Bosch and Pol, 2022). Although historically presumed to be innocuous and inert lipid storage depots, many studies now highlight the dynamic nature of LDs in terms of their formation, turnover, and composition across a number of homeostatic and pathological conditions, including neurodegenerative disease (Farmer et al., 2020). Both the lipid content and outer protein profile of a LD can vary greatly, with wide-ranging consequences for cellular function. For example, LDs liberate fatty acids as lipid mediators of inflammation (Khor et al., 2014) or for mitochondrial metabolism (Jarc and Petan, 2020). Differences in either the inner neutral lipid content or the phospholipid-rich shell can influence the assembly and function of proteins on the LD monolayer (Walther and Farese Jr., 2012).

A set of ubiquitous LD proteins known as perilipins (PLINs) provide clues about LD function based on their expression within the cell. For example, PLIN5 is associated with oxidative phosphorylation and colocation of LDs to the mitochondria (Bosch and Pol, 2022; Gallardo-Montejano et al., 2021; Wang et al., 2011; Kien, 2022; Miner et al., 2023), whereas PLIN2 expression is associated with immunologic and inflammatory function within the cell (Bosch et al., 2020; He et al., 2022). With this information in mind, it is reasonable to believe the LD composition and protein makeup is as dynamic as their now recognized multitude of functions and should be considered when studying LD accumulation in disease. It is well documented that several diseases are associated with excessive LDs, including but not limited to obesity, atherosclerosis, cancer, type 2 diabetes, and Alzheimer’s disease (AD) (Farmer et al., 2020; Dalhaimer, 2019); and evidence suggests these organelles may be active participants in disease mitigation, progression, or both (Onal et al., 2017).

Genome-wide association studies (GWAS) identify many late-onset Alzheimer’s disease (LOAD) risk genes involved in the immune response and microglia function (Griciuc and Tanzi, 2021), particularly genes involved in microglial phagocytosis and debris processing (Podlesny-Drabiniok et al., 2020). The strongest of the LOAD risk genes is the ε4/ε4 (E4) allele of Apolipoprotein E (*APOE*), a gene also strongly implicated in microglial function (Krasemann et al., 2017; Lee et al., 2023; Yin et al., 2023). *APOE* is a disease-associated-microglia (DAM) gene, and diseased or stressed microglia upregulate production of ApoE (Krasemann et al., 2017). Intriguingly, ApoE is also droplet-associated protein found on the LD surface (Liu et al., 2023; Bersuker et al., 2018) and E4 expressing astrocytes (Windham et al., 2024; Farmer et al., 2019; Sienski et al., 2021) and E4 microglia (Machlovi et al., 2022; Victor et al., 2022; Haney et al., 2024) both accumulate more lipid droplets than neutral AD-risk ε3/ε3 (E3) expressing cells. Additionally, carriage of the E4 allele is associated with increased neuroinflammation and metabolic disturbances within neurons and glial cells (Krasemann et al., 2017), two functions potentially altered by LD accumulation and composition.

It is of particular interest that glial cells accumulate LDs in response to neuronal stress, age, or neurodegeneration (Farmer et al., 2020; Ioannou et al., 2019; Marschallinger et al., 2020). LD accumulation has been described as both a beneficial compensatory response and a pathological feature within the brain. For example, there is compelling evidence of a synergistic relationship between neurons and astrocytes whereby fatty acids are shuttled from neurons to sequester in astrocytic LDs to protect neurons against fatty acid toxicity (Ioannou et al., 2019). However, a more pathologic accumulation of LDs associated with age in microglia causes abnormal cellular function (Ioannou et al., 2019). Lipid accumulation with age is highly relevant in Alzheimer’s disease (AD), where even the first description of AD by Alois Alzheimer noted glia with adipose inclusions (Alzheimer et al., 1995).

While there are numerous reports of both peripheral and CNS cell types accumulating more LDs in the presence of E4, the effects of *APOE* on the composition of LDs themselves remains unexplored (Farmer et al., 2019; Sienski et al., 2021; Machlovi et al., 2022; Victor et al., 2022; Haney et al., 2024; Qi et al., 2021; Johnson et al., 2011; Huang et al., 2022; Gluchowski et al., 2017). Here, we sought to develop a deeper knowledge of LD characteristics between *APOE3 and APOE4* expressing cells in the liver and brain. E3 and E4 expressing LDs were analyzed for their relative accumulation at baseline and in response to an inflammatory challenge, their physical characteristics (including size, lipid, and protein composition), and their potential effects on microglia function.

## 2. Results

### 2.1. LD-enriched fractions show greater droplet accumulation in E4 and inflamed conditions

**Fig. 1.**
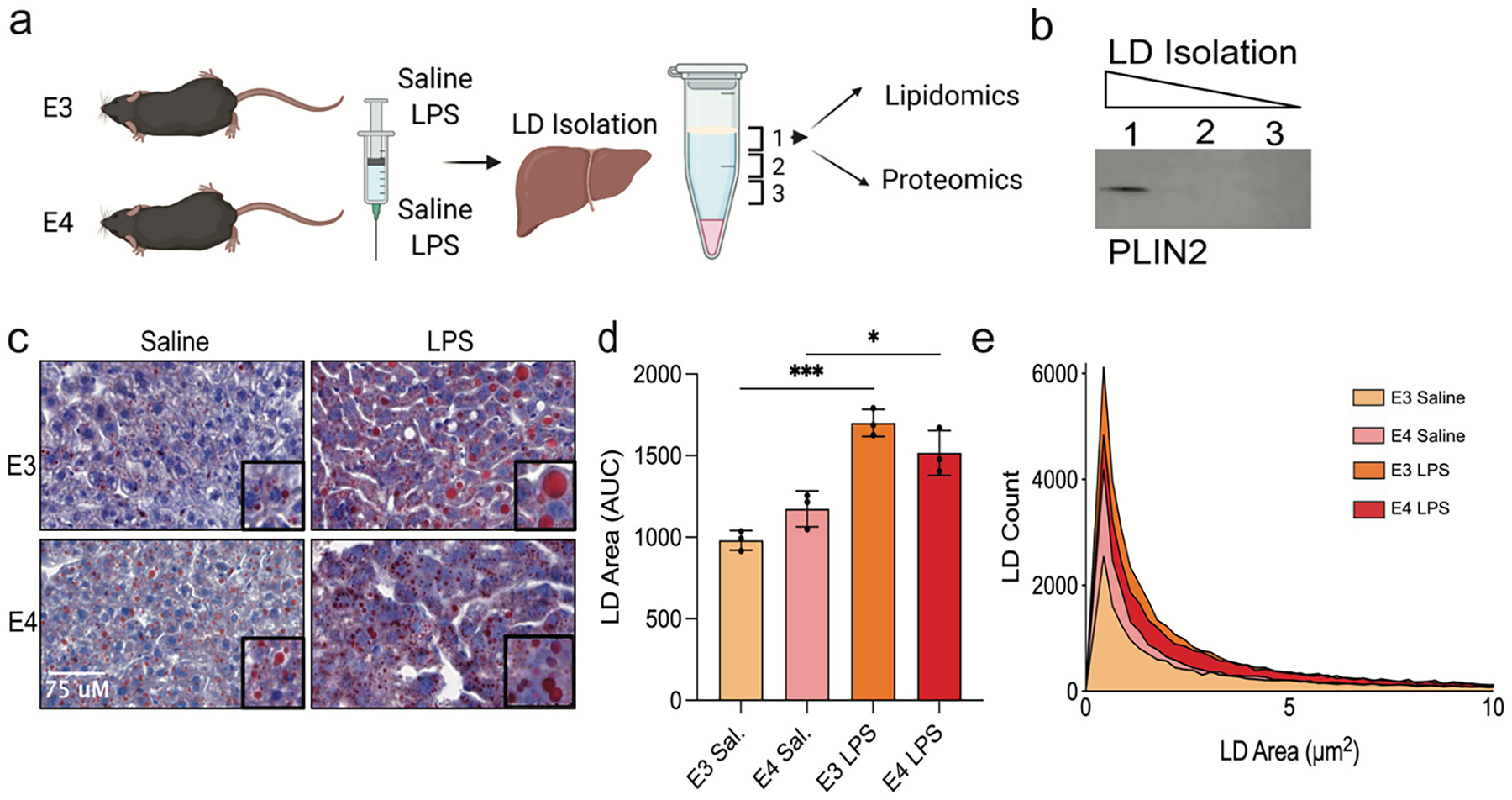
Lipid droplet isolation from mouse liver tissue successfully targeted the droplet fraction and inflamed liver tissue showed increased lipid via ORO stain. a) Targeted replacement mice expressing human ApoE3 or ApoE4 (*n* = 5 per group) were treated with either saline or 5 mg/kg LPS intraperitoneally. After 24 h, liver tissue was collected for lipid droplet (LD) isolation, proteomics, and lipidomics. b) Western blot of sequential fractions from the density gradient confirmed successful LD isolation, with the LD marker PLIN2 present only in the top layer. c) Liver sections were stained with Oil Red O (ORO) to visualize neutral lipid accumulation (*n* = 3 per group). d) Quantification of ORO-stained area (AUC) was analyzed using two-way ANOVA, which revealed a significant interaction between genotype and treatment (F(1,8) = 10.19, *p* = 0.0128), a main effect of treatment (F(1,8) = 81.37, *p* < 0.0001), and no significant main effect of genotype (F(1,8) = 0.006, *p* = 0.939). Post hoc Tukey’s test: *p < 0.05, ***p < 0.001. e) Histogram displaying the distribution of LD sizes based on ORO staining across groups.

Extracting sufficient LDs from microglia – either in vitro or in vivo – is prohibitive due to their relatively small LD content compared to the large quantities of cellular material necessary for successful LD isolation and quantitative ‘omics. Therefore, we first studied the more abundant and easily accessible LDs from the liver of mice expressing human E3 or E4 to quantify ApoE isoform-specific changes in LD composition (Fig. 1a). Mice were injected with saline or LPS (5 mg/kg) and liver tissue was harvested after 24 h to capture early changes in LD dynamics. LDs were enriched via density gradient centrifugation where the buoyant, lipid-rich fractions at the top of the centrifuge tube were collected for western blot verification of lipid droplet surface proteins PLIN2, PLIN3, and PLIN5 (Fig. 1b, Supplemental Fig. 1). Both E3 and E4 liver tissue increased lipid content after LPS stimulation, but a significant interaction effect (F (1,8) = 10.19; *p* = 0.0128) suggests this response more robust in E3 LPS livers compared to E4 LPS livers (Fig. 1c–d). Together, these data validate an *APOE*-specific, LD-enriched source material for downstream analysis of the proteome and lipidome.

### 2.2. E4- and LPS-stimulated LDs are enriched in phosphatidylcholine

**Fig. 2.**
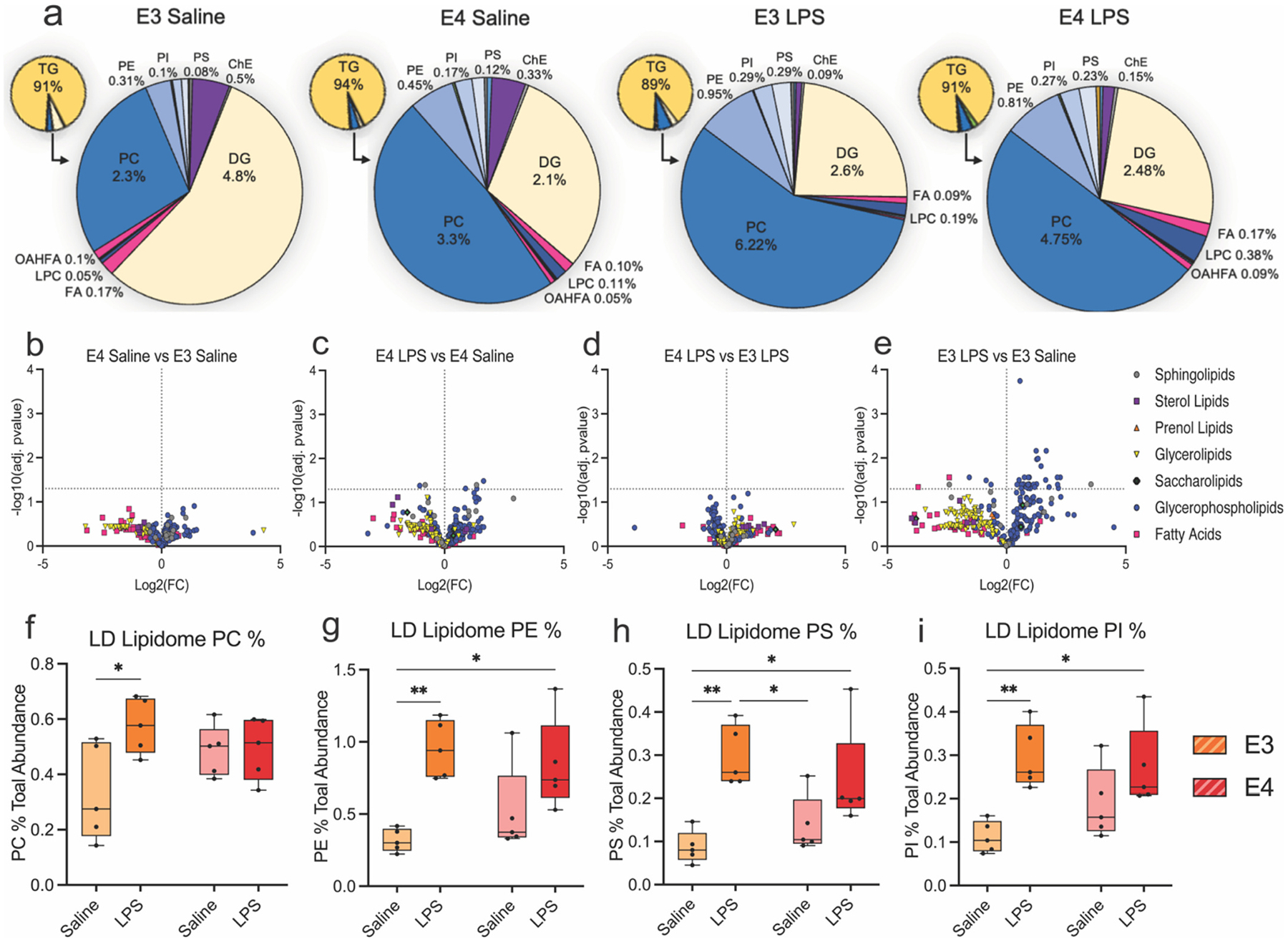
The lipidome of E4 lipid droplets at baseline resembles that of LPS-treated droplets. a) Pie charts showing the distribution of lipid classes within lipid droplets across genotype and treatment groups. Small pie charts highlight the relative abundance of triacylglycerol (TG), while larger pie charts display the composition of remaining lipid species. b–e) Volcano plots of differentially abundant lipid classes between groups. b) E4 vs. E3 saline: lipids elevated in E4 saline appear on the right. **c)** E4 vs. E3 LPS: highlights shift in lipid class abundance following LPS in each genotype. d–e) Within-genotype comparisons between saline and LPS-treated droplets for E3 (d) and E4 (e) groups. f–i) Box and whisker plots of individual phospholipid classes showing relative abundance (show as % of total lipid abundance) across groups: f) phosphatidylcholine (PC), g) phosphatidylethanolamine (PE), h) phosphatidylserine (PS), and i) phosphatidylinositol (PI). For f-i, Two-way ANOVA with Tukey’s post hoc test was used to assess significance. *p < 0.05, **p < 0.005. Statistical values for each test are reported in Supplementary Table, Table 1.

As anticipated, the bulk of lipid content in LD enriched fractions was composed of glycerolipid, with ~90 % of the lipidome consisting of triacylglycerols (TGs), a common neutral lipid component of the LD inner core (Fig. 2a, Supplemental Table 1, Table 1) (Olzmann and Carvalho, 2019). The other lipid species detected were largely neutral diacylglycerols (DGs) and glycerophospholipid species that make up the outer LD monolayer (Fig. 2a, large circles). Phosphatidylcholine (PC) was notably enriched in E4 compared to E3 LDs at baseline, as well as in LPS treated groups (Fig. 2a). Similarly, volcano plots of differentially abundant metabolites show that while E3 droplets increase their PC content when stimulated with LPS, a similar response is not observed in E4 droplets (Fig. 2b–e). A comparison of the E3 Saline to E3 LPS LD lipidome showed significant increase in the percentage of PC, PE, PI, and PS; however, these same LPS-dependent increases were not observed in the E4 LD lipidome (Fig. 2f–i). A similar trend was seen in the saturation of lipid acyl chains on TGs and cholesterol esters (ChE), where the abundance of polyunsaturated fatty acids (PUFA) and monounsaturated fatty acids (MUFA) are significantly decreased between E3 saline and E3 LPS, but no change is observed between the E4 groups (Supplemental Fig. 2). Finally, the total abundance of ChE content decreased more robustly in E3 LDs following LPS treatment compared to the E4 LDs. These results suggest that unlike E3 LDs, which substantially remodel their lipidome in response to an inflammatory challenge, E4 LDs show a blunted response and display an intermediate lipid profile between baseline E3 and the post-inflammatory state.

### 2.3. E4 droplets are enriched with TG sequestration proteins, depleted in fatty acid β-oxidation proteins

**Fig. 3.**
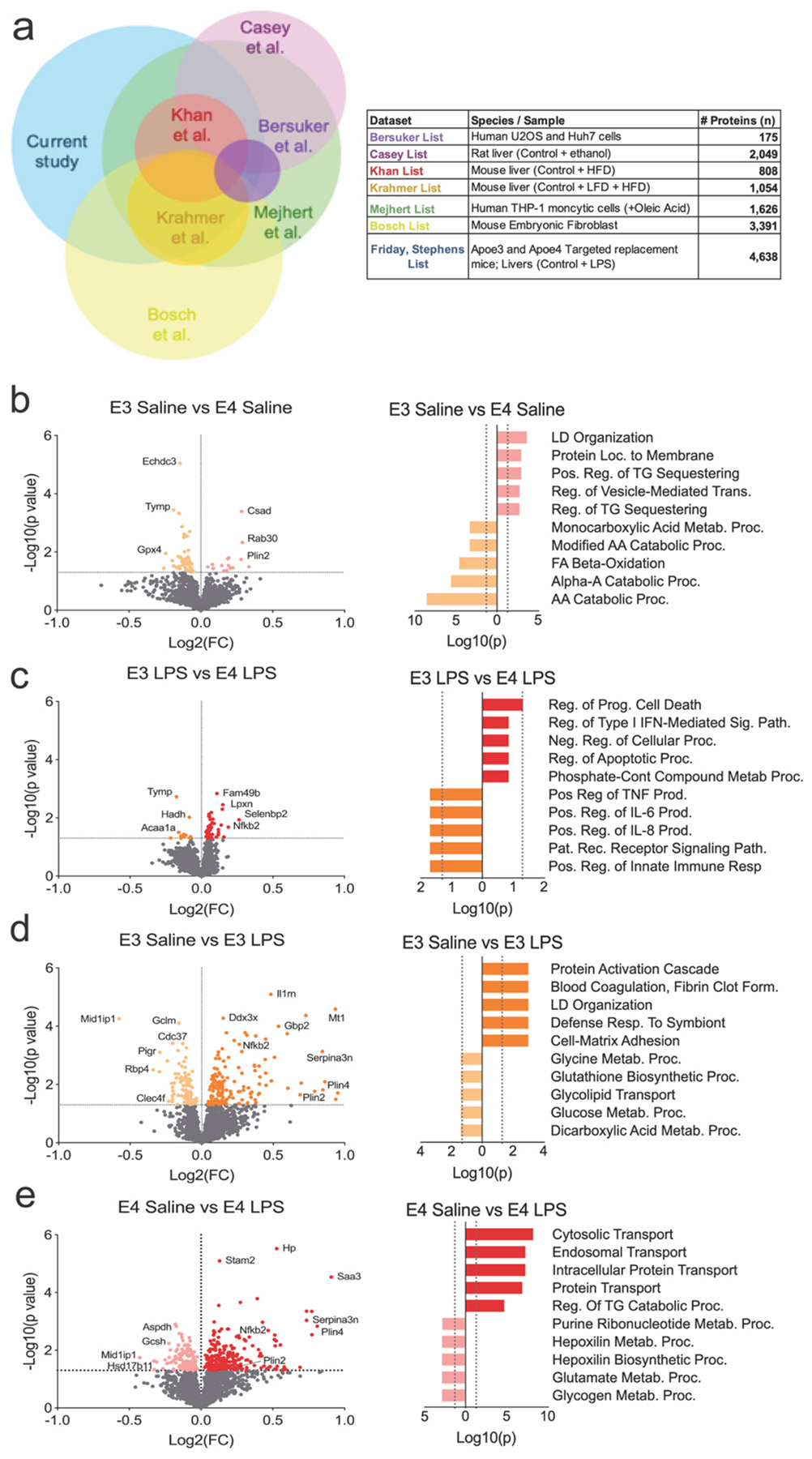
Differentially expressed proteins and gene ontology analyses reveal distinct metabolic and immune pathways in APOE3 vs. APOE4 lipid droplets. a) Venn diagram showing overlap between the APOE lipid droplet (LD) proteome from the current study and previously published datasets. Table summarizes the number of proteins identified in each study and their respective sources. b–e) Volcano plots (left) display differentially expressed proteins (DEPs) between indicated conditions; corresponding gene ontology (GO) enrichment plots (right) show biological processes enriched among significantly upregulated proteins. b) At baseline (saline), APOE3 LDs are enriched for proteins involved in lipid droplet organization, fatty acid β-oxidation, and amino acid metabolism compared to APOE4 LDs. c) Following LPS stimulation, APOE3 vs. APOE4 LDs differ in proteins related to apoptosis and immune signaling, including TNF and NF-κB pathways. d) Within APOE3 LDs, LPS increases proteins involved in cytokine signaling, immune activation, and cholesterol metabolism. e) Within APOE4 LDs, LPS drives enrichment of proteins associated with vesicle transport, ER trafficking, and some metabolic functions, though lipid-related terms are less prominent than in E3. **Abbreviations:** AA, amino acid; FA, fatty acid; Loc, location; Neg, negative; Pat, pattern; Pos, positive; Proc, process; Rec, receptor; Reg, regulation; TG, triglyceride.

Quantitative proteomic analyses of the LD-containing fractions revealed at total of 4638 LD-enriched proteins (Supplementary Table 1, Table 2). We cross referenced these with other known LD proteomes to identify proteins that are consistently and/or exclusively expressed on the LD surface (Fig. 3a, Supplementary Table 1, Table 3), which resulted in a total of 2338 ‘LD resident’ proteins which overlapped with at least one other dataset (Bosch et al., 2020; Bersuker et al., 2018; Mejhert et al., 2020; Khan et al., 2015; Krahmer et al., 2018; Casey et al., 2021). Gene ontology analyses showed that under basal conditions, E4 LDs were enriched for proteins involved in LD organization, membrane trafficking, and TG sequestration, while they were relatively depleted in proteins related to fatty acid β-oxidation (Fig. 3b). Following LPS treatment, a comparison of the proteome between E4 and E3 LDs reflected differences in the type of immune proteins localized to the LD surface (Fig. 3c), while a within-genotype comparison of the LPS response highlighted changes in LD proteins related to reorganization and transport, as well as several metabolic pathways (Fig. 3d–e). We further utilized our proteome data set to analyze pathway enrichment between our treatment and genotype comparisons. Within genotypes, LPS caused enrichment of similar pathways in both E3 and E4 LDs. Specifically, this included enrichment of interferon response and interlukin-6 signaling pathway (Supplemental Fig. 3c–d, Supplementary Table 1, Table 8). Interestingly, E3 LDs showed enrichment for fatty acid metabolism and oxidative phosphorylation pathways in both saline and LPS treatment when compared to E4, which showed enrichment in inflammatory response and complement pathway in the saline or LPS comparison respectively (Supplemental Fig. 3a–b, Supplementary Table 1, Table 8).

**Fig. 4.**
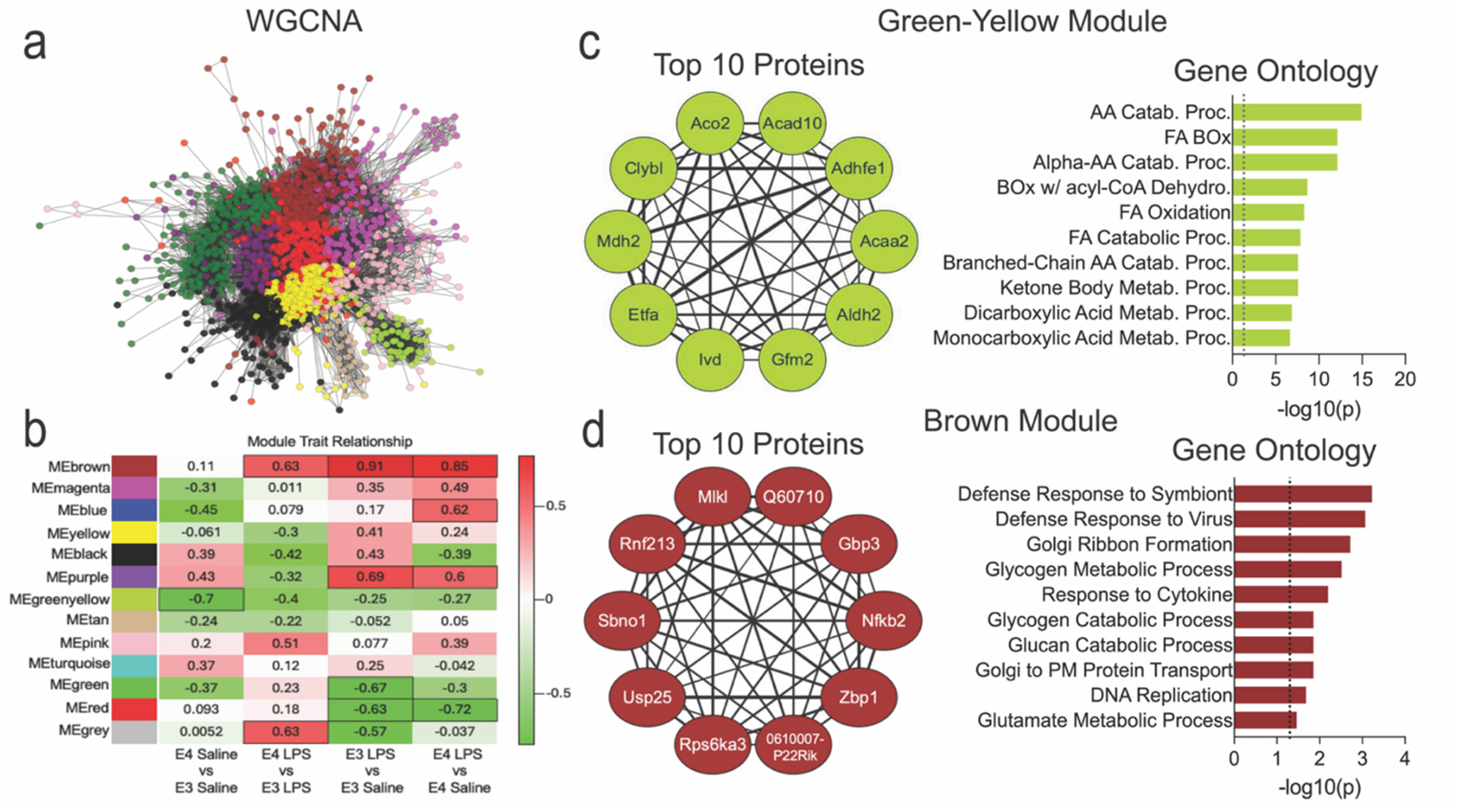
WGCNA analysis reveals significant differences in fatty acid metabolism and vesicle transport pathways between E3 and E4 lipid droplets. a) Whole-network visualization of weighted gene co-expression network analysis (WGCNA) across the LD proteome. b) Heatmap of eigengene-trait correlations for 13 WGCNA modules, comparing E3 and E4 lipid droplets under saline and LPS treatment. Outlined boxes denote statistically significant associations (*p* < 0.05). c) The green-yellow module showed one of the strongest genotype-dependent differences, with significantly lower expression in E4 LDs. GO enrichment revealed involvement in lipid metabolic processes, including fatty acid β-oxidation, branched-chain amino acid catabolism, and TCA-related pathways. d) The brown module was upregulated by LPS treatment, particularly in E4 microglia, and enriched for immune response, glycogen metabolism, and vesicle trafficking pathways. In panels (c–d), network diagrams display the top 10 hub proteins (highest kME), and the adjacent bar plots represent GO enrichment based on all proteins within each module. **Abbreviations:** AA, amino acid; FA, fatty acid; β-ox, β-oxidation; Catab., catabolism; Proc., process.

To identify potential protein networks, we next performed a weighted gene co-expression network analysis (WGCNA) of the LD proteome (Fig. 4a–b, Supplementary Table 1, Table 4). Among these, the green-yellow module was significantly downregulated in E4 LDs at baseline and was enriched for metabolic pathways, including fatty acid β-oxidation, branched-chain amino acid catabolism, and the TCA cycle (Fig. 4c). Notably, the top hub proteins within this module, such as Aco2, Acaa2, Mdh2, Etfa, and Aldh2, are mitochondrial enzymes critical for oxidative metabolism and energy production, suggesting that E3 microglia maintain a more catabolic, oxidative lipid metabolic state, while E4 microglia exhibit reduced mitochondrial engagement even in the absence of stimulation. In contrast, the brown and purple modules were upregulated by LPS across genotypes and enriched for processes including innate immune response, glycogen metabolism, and protein or vesicle trafficking (Fig. 4d, Supplemental Fig. 4). The brown module, in particular, featured hub proteins like Nfkb2, Gbp3, and Rnf213, consistent with a transcriptional and antiviral response. In sum, these results show that i) the E3 vs E4 response to an inflammatory challenge features varying LD protein dynamics, and ii) the E4 LD is enriched for proteins that facilitate lipid storage over utilization.

### 2.4. Proteins previously tied to APOE4 and AD are largely LD-enriched

**Fig. 5.**
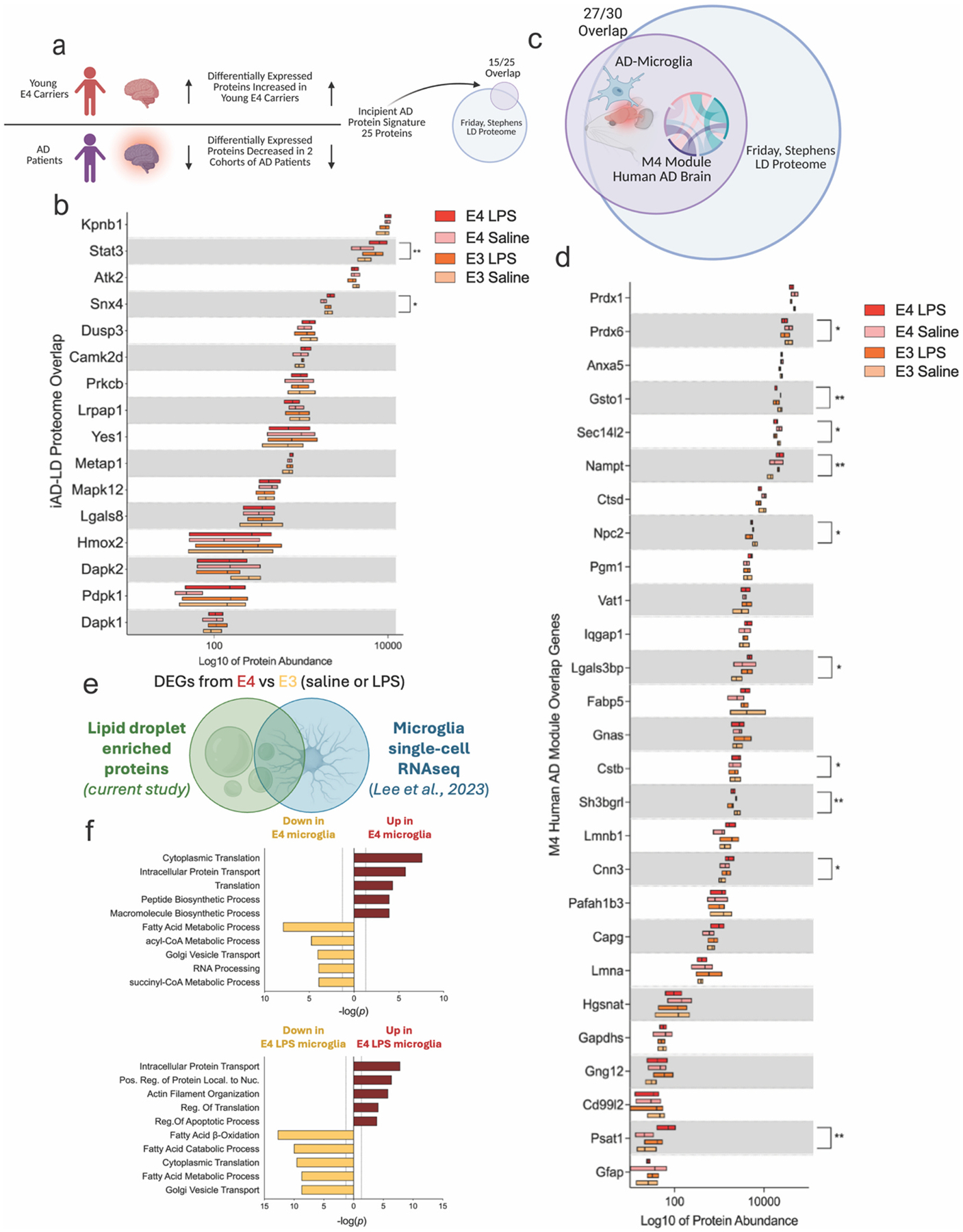
The lipid droplet (LD) proteome significantly overlaps with human Alzheimer’s disease (AD) and microglial metabolism signatures. a) A prior study by Roberts et al. identified an “incipient AD” (iAD) protein signature, where proteins were increased in young APOE4 carriers but decreased in AD brains. This iAD signature showed 60 % overlap with the APOE LD proteome from the current study. b) Protein abundance of overlapping iAD proteins across E3 and E4 microglial LDs. Two-way ANOVA: interaction F(45, 256) = 3.754, *p* < 0.0001; row factor F(15, 256) = 716.8, p < 0.0001; column factor F(3, 256) = 5.323, *p* = 0.0014. *p < 0.05, **p < 0.005. c) A proteomic study by Johnson et al. defined an AD-associated “M4” glial metabolism module, which was differentially expressed in human AD brains and in microglia from a mouse model of AD. This M4 module shared 90 % overlap with our LD proteome. d) Protein abundance of overlapping M4 glial metabolism proteins across groups. Two-way ANOVA: interaction F(78, 432) = 3.632, p < 0.0001; row factor F(26, 432) = 818.6, p < 0.0001; column factor F(3, 432) = 4.510, *p* = 0.0040. e) Overlap between LD-enriched proteins and differentially expressed genes (DEGs) from E3 vs. E4 microglia (saline or LPS) in a previously published single-cell RNA-seq dataset (Lee et al., 2023). f) Gene ontology enrichment of LD-enriched proteins differentially expressed between E3 and E4 liver LD proteomes and differentially expressed between E3 and E4 microglia. In E4, these overlapping proteins were enriched for pathways related to protein synthesis and transport and downregulated for fatty acid metabolism.

The LD proteome analyses above revealed several proteins that we recognized from the AD literature. To systematically assess this, we mined several proteomic and transcriptomic datasets for comparison, including a unique protein signature referred to as “incipient AD” (iAD) (Roberts et al., 2021). The iAD signature is a set of proteins highly expressed in young E4 carrier brains but reduced in AD brains (Roberts et al., 2021). Intriguingly, 15 out of 25 (60 %) of these AD-predictive proteins were highlighted in our LD proteome (Fig. 5a–b, Supplemental Fig. 5). Another proteomic analyses of human AD brain identified a network module related to microglial metabolism (termed “M4”) (Johnson et al., 2020). In that study, Johnson et al. characterized the top 30 most differentially expressed microglial transcripts from an AD mouse model that corresponded to M4. Notably, this list showed near complete overlap (27 of 30 proteins) with our LD proteome (Fig. 5c–d, Supplemental Fig. 6). Similar results can be seen from a comparison with Dai et al. where ~35 % of proteins upregulated in E4/4 AD compared to E3/3 AD brains (Dai et al., 2018) overlapped with proteins enriched on E4 LDs. In contrast, ~29 % of proteins upregulated in E3/3 AD compared to E4/4 AD brains overlapped with proteins enriched on E3 LDs and were associated with pathways of fatty acid β-oxidation (Supplementary Table, Table 3). We next overlaid our list of LD-enriched proteins with differentially expressed genes in microglia from E3- or E4-expressing mice treated with either saline or LPS (Lee et al., 2023). Specifically, proteins which were higher in our E4 saline livers (compared to E3 saline) and overlapped with genes higher in saline treated E4 microglia (compared to E3 saline, 5.4 % of the LD-enriched protein overlap with microglia DEGs) were used to run gene ontology enrichment. This process was repeated for the E3 saline (3.6 % overlap), E4 LPS (4.6 % overlap), and E3 LPS groups (4.1 % overlap). In both the saline and LPS treated conditions, the overlap of LD proteins and microglial genes differentially regulated by E4 pointed to an upregulation of pathways of protein synthesis and transport along with a concomitant downregulation of fatty acid metabolism (Fig. 5e–f). Additionally, we confirmed via immunocytochemistry that select proteins from our LD-microglia overlap (Apoe, SNX4, and Pla2g4a) co-localized with LDs in our primary microglia cultures (Supplemental Fig. 7). Together, these data show the protein composition of liver-derived LDs – and the effect of E4 in modulating enrichment of these proteins – substantially overlaps with the established E4/AD proteome and are present on microglia LDs, suggesting that many of the same proteins that define AD pathogenesis are found on or closely associated with LDs.

### 2.5. E4 microglia accumulate more LDs and secrete more cytokines following a variety of stimuli

**Fig. 6.**
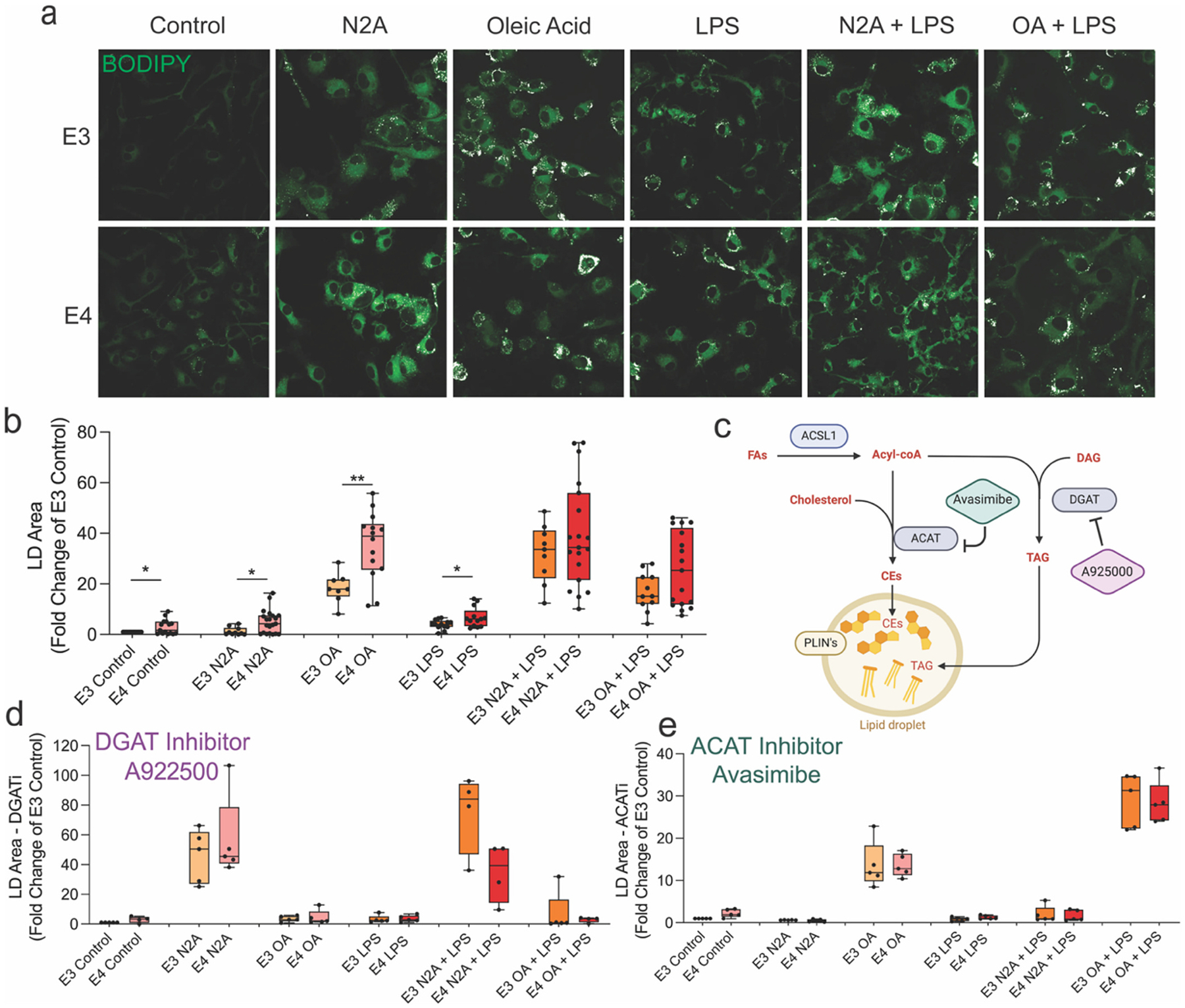
E4-expressing microglia accumulate more lipid droplets than E3 microglia under multiple stimuli; DGAT and ACAT inhibitors reduce droplet formation in both genotypes. a–b) Primary microglia from ApoE3 or ApoE4 mice were treated with oleic acid (OA), lipopolysaccharide (LPS), necrotic N2A cells (nN2A), or combinations of these stimuli. Lipid droplets (LDs) were labeled using BODIPY 493/503. E4 microglia showed significantly greater LD accumulation than E3 microglia under control conditions and after OA, nN2A, or LPS treatment. c) Schematic of the metabolic pathways targeted for LD inhibition. DGAT inhibition (A922500) blocks triacylglycerol (TAG) synthesis, while ACAT inhibition (Avasimibe) blocks cholesterol ester (CE) formation from cholesterol and free fatty acids. d) DGAT1 inhibition reduced LD accumulation in both genotypes across most conditions, except following nN2A treatment. e) ACAT inhibition reduced LD formation in LPS- and nN2A-treated cells but was less effective in OA-treated microglia. Statistical significance was determined by multiple unpaired *t*-tests with a 5 % false discovery rate (FDR) correction. *p < 0.05, **p < 0.01.

Given the ostensible AD-microglia signature in the LD proteome, we examined LDs in E3 and E4 primary mouse microglia. Microglia were treated with exogenous fatty acid (OA), LPS, necroptotic N2A cells (nN2A), or combinations thereof, and lipid droplet (LD) formation was assessed at 24 h using BODIPY staining (Fig. 6a–b). Under baseline, OA alone, nN2A alone, and LPS alone, E4 microglia accumulated significantly more LDs than E3 (E4 vs. E3: *p* < 0.05 for all). However, in the combined-stimulus conditions (OA + LPS and nN2A + LPS), the E4–E3 difference was no longer significant. To evaluate the contribution of specific LD synthesis pathways, cells were treated with either the DGAT1 inhibitor A922500 (Teixeira et al., 2023) or the ACAT1 inhibitor Avasimibe (Zhu et al., 2021). DGAT1 inhibition robustly suppressed LD accumulation across all conditions except nN2A, suggesting that neuronal debris–derived lipids are likely routed into alternative esterification pathways, such as ACAT-mediated cholesterol esterification, phospholipid remodeling via the Lands’ cycle, and/or DGAT2-derived TAG synthesis (Fig. 6d, Supplemental Fig. 8). In contrast, ACAT1 inhibition significantly reduced LD formation in all conditions except OA, consistent with the role of fatty acids in driving DGAT-dependent neutral lipid accumulation (Fig. 6e, Supplemental Fig. 9). Representative images of inhibitor-treated cells are shown in Supplemental Figs. 8 and 9. A summary comparison of droplet area across all conditions is provided in Supplemental Fig. 10.

**Fig. 7.**
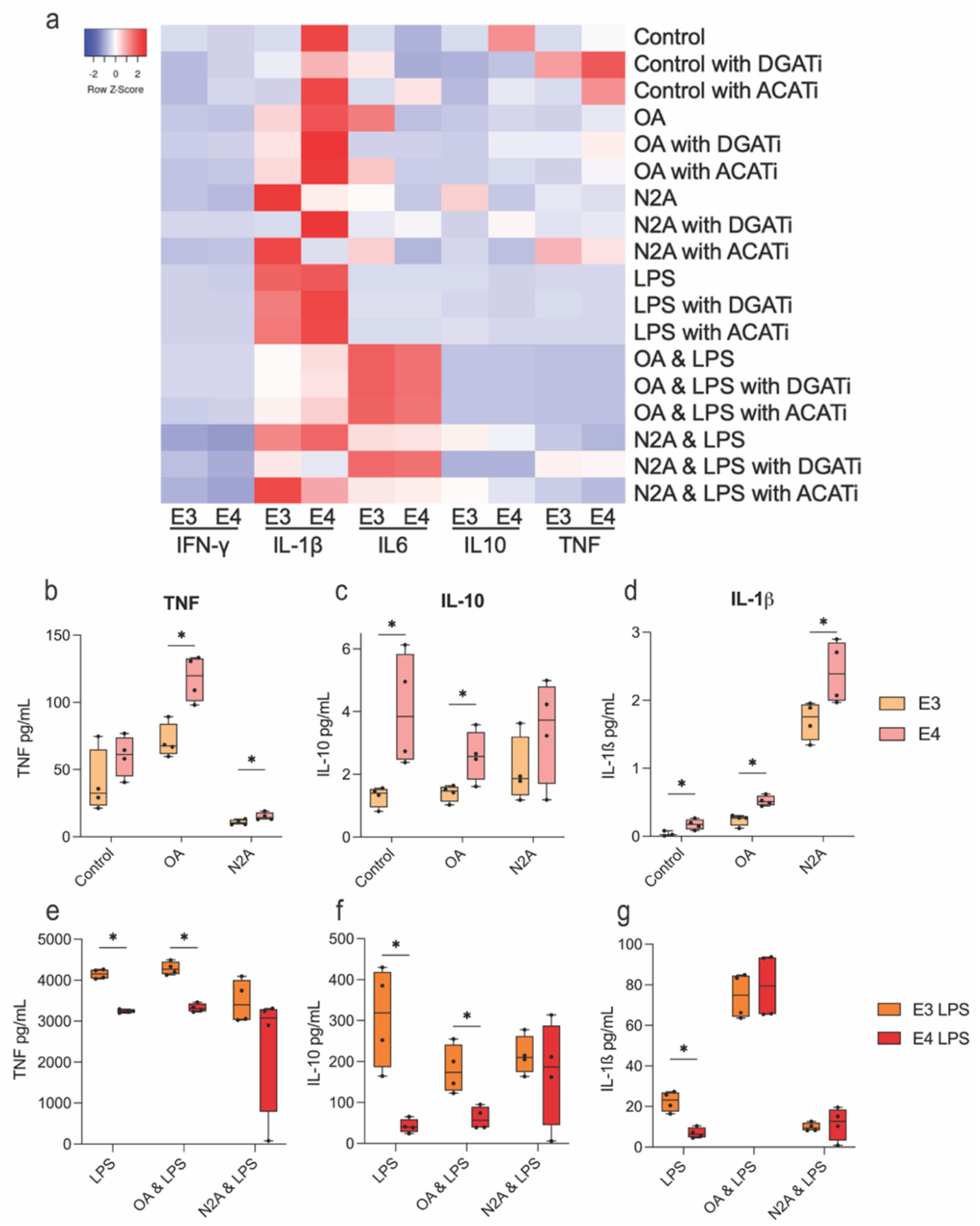
E4 microglia secrete more cytokines at baseline but exhibit a blunted cytokine response to LPS; inhibition of lipid droplet (LD) formation does not attenuate cytokine release. a) Heatmap of cytokine levels in the culture media of E3 and E4 microglia following treatment with OA (oleic acid), necrotic N2A cells (nN2A), or LPS, with or without inhibition of LD formation using DGAT or ACAT inhibitors. b–d) Cytokine levels (TNF, IL-10, IL-1β) in the media of E3 and E4 microglia treated with OA or nN2A alone (“baseline” conditions). e–g) Cytokine levels (TNF, IL-10, IL-1β) following co-treatment with OA or nN2A plus LPS stimulation. Statistical significance was assessed by multiple unpaired *t*-tests with a 5 % false discovery rate (FDR) correction.

Lipids within LDs can be liberated to become mediators of inflammation, and in aging and AD, lipid droplets accumulate in microglia and cytokine production increases (Jarc and Petan, 2020; Haney et al., 2024; Marschallinger et al., 2020). Measurement of cytokine release into the media showed that E4 microglia secreted more TNF, IL-1β and IL-10 than E3 at baseline, as well as when exposed to OA. In response to nN2A, E4 secreted more TNF and IL-1β, but not IL-10. (Fig. 7a–d). As expected, the addition of LPS resulted in a dramatic increase in the release of these cytokines in control, OA, and nN2A treated cells; however, this effect was blunted in E4 microglia for TNF and IL-10 (Fig. 7e–g). Notably, while blocking ACAT1 lowered IFN-γ secretion, particularly in E4 microglia, preventing LD formation by inhibiting either ACAT1 or DGAT did not decrease cytokine output as expected (Fig. 7a, Supplemental Table 1, Table 5 & Figs. 11–12). While some selective cytokine modulation was observed our results suggest that inhibition of LD formation is largely insufficient to reduce cytokine production. Together, these data suggest that E4-expressing microglia accumulate more LDs and secrete higher levels of cytokines at baseline, findings in line with other recent studies (Machlovi et al., 2022; Victor et al., 2022; Haney et al., 2024; de Leeuw et al., 2022). However, the response of E4 microglia to these various stimuli has proven to be nuanced and deserving of further investigation.

## 3. Discussion

In the current study, we provide the first *APOE* genotype-specific characterization of the lipid droplet-ome. We show that LDs from humanized E4 mice have a distinct lipid and protein profile compared to E3 droplets, both at baseline and following an acute inflammatory challenge. Building upon a growing literature, we also show that E4 expressing microglia accumulate more LDs and secrete more cytokines following various stimuli. Finally, by comparing our APOE-LD proteome with previous ‘omics analyses of postmortem AD/E4 brain and AD/E4 microglia, our data suggest that many of these previously implicated proteins are LD-associated.

Rather than inertly sequestering lipid, it is now appreciated that LDs play a much more active role within the cell, working to integrate metabolism and the immune response by hosting a number of different proteins on their membranes. One of these proteins recently shown to traffic to the LD surface is ApoE (Windham et al., 2024), an apolipoprotein whose common variants impart dramatically different risk for AD (Reiman et al., 2009; Fortea et al., 2024). Our findings of more LDs in E4-expressing microglia are in agreement with a growing number of studies showing a disproportionate buildup in E4 compared to E3 livers (Johnson et al., 2011), fibroblasts(Tambini et al., 2016), astrocytes (Farmer et al., 2019; Sienski et al., 2021; Qi et al., 2021; Tcw et al., 2022), and microglia (Machlovi et al., 2022; Victor et al., 2022; Haney et al., 2024). Additionally, a number of studies have examined the effects of *APOE* genotype on the lipid profile of glia in vitro (Windham et al., 2024; Sienski et al., 2021; Qi et al., 2021). However, these previous studies did not address a possible effect of *APOE* on the composition lipid droplets themselves. In our view, the connection between ApoE itself as a droplet resident protein, ApoE’s role in lipid trafficking and metabolism, and E4’s role as the largest genetic risk factor for late-onset AD implored further investigation into LD dynamics between *APOE* genotypes.

Lipidomic analysis of our isolated LD fractions indicated that the droplets were primarily composed of TGs, a finding expected of droplets in the liver. However, other lipids within the droplet core and membrane differed markedly by genotype and treatment condition. For example, several phospholipid species were altered and, perhaps surprisingly, the abundance of cholesterol was lower in our LPS samples. At baseline, E4-LDs showed an enrichment in PC that resembles the lipid profile of LPS-treated droplets. This finding could be indicative of a smaller droplet size and increased surface-to-volume ratio that would require increased phospholipids in the membrane. Specifically, PC incorporation in the LD monolayer has been shown to act as a surfactant to prevent LD coalescence (Krahmer et al., 2011), and an increased surface-to-volume ratio of smaller droplets may provide clues to their fate within the cell; i.e. larger droplets are more prone to lipolysis for fatty acid liberation to contribute to β-oxidation, while smaller droplets are more prone to lipophagy (Schott et al., 2019). In line with this, our lab previously described smaller LDs in E4 astrocytes along with an E4-associated shift away from FA oxidation and toward aerobic glycolysis (Farmer et al., 2019; Farmer et al., 2021). This line of thinking is bolstered by the proteomics gene ontology and WGCNA results which showed a depletion of FA β-oxidation proteins on the E4 LDs relative to E3.

Regarding the LD proteome, an exciting finding from our profiling of LD-enriched liver fractions was a surprisingly high overlap with multiple AD/APOE4/microglia proteomic and transcriptomic datasets. For example, we found significant overlap of our LD-enriched protein list with an “incipient AD” proteomic signature of young E4-carriers published by Roberts et al. In this study, tissue from two human AD studies were analyzed for proteomic differences between cognitively normal and AD brains. Differentially expressed proteins from both studies were compared to significantly changed proteins in the brains of young E4-carriers and non-E4-carriers. This revealed a set of 25 proteins that were intriguingly upregulated in young E4 carriers yet downregulated in AD. Interestingly, 15 of the 25 identified proteins are LD-proteins from our database (with 9 of these confirmed in other LD databases). Several of these proteins have been previously implicated in AD pathogenesis. For example, the inflammatory transcription factor STAT3 and sorting nexin (SNX4) are of particular interest based on their contribution to inflammation and amyloid beta accumulation, respectively (Toral-Rios et al., 2020; Kim et al., 2017). Further investigation of how these proteins – and others identified here – contribute to LD-related function and AD pathology is warranted.

Another noteworthy overlap in the LD-proteome is with data from a large-scale proteomics analysis of AD postmortem tissue (Johnson et al., 2020). There, Johnson et al. identified a network module (“M4”) of putative microglial metabolism proteins that shared ontology with microglial genes from an AD mouse model (Johnson et al., 2020). Of these thirty M4 network proteins, we found 27 to be LD-enriched, and these proteins include previously established DAM markers such as Lgals3 and Fabp5 (Krasemann et al., 2017). While that particular study did not include *APOE* genotype in its analysis, a previous study from the Seyfried laboratory highlighted differentially expressed proteins from E4-expressing brain tissue (Dai et al., 2018). Again, our LD-enriched proteome showed high overlap, with around one third of the proteins up-regulated in the E4 AD (or E3 AD) brain also being upregulated on the surface of E4 (or E3) LDs. Finally, differentially expressed genes in microglia from E3- or E4-expressing mice treated with either saline or LPS (Lee et al., 2023) also showed substantial overlap with our LD proteome, again highlighting the potential role of *APOE* and LDs within this critical immune cell. Interestingly, in each case above, gene ontology of these shared LD-AD proteins pointed toward a depletion of FA β-oxidation on E4 relative to E3 LDs. As from several previous studies, this highlights a potential pathogenic role of E4 in modulating glial FA metabolism (Farmer et al., 2019; Sienski et al., 2021; Machlovi et al., 2022; Victor et al., 2022; Haney et al., 2024; Qi et al., 2021) with the data here implicating LDs as the initiating hub.

Though we performed lipidomics and proteomics analyses on LDs isolated from the liver rather than brain by necessity, this strong overlap with previous AD-microglia proteomic and transcriptomic datasets supported a translation of these findings. In fact, similar trends regarding the effects E4 were seen between our LD proteome and differentially expressed genes from scRNA sequencing of microglia isolated from the brains of humanized APOE mice subjected to the same LPS paradigm. This suggest that E4s influence on LDs could be cell type agnostic, but future analysis on overlap in other cell types within the CNS, like neurons and astrocytes, may provide further insight into the cell type dependent and independent effects of *APOE* on LD regulation. Additionally, a handful of studies have inhibited LD formation and observed rescue of some abnormal cellular activity as a result. For example, Marshallinger et al. observed increases in pro-inflammatory cytokines, ROS, and abnormal function in LD-accumulating microglia (LDAM) (Marschallinger et al., 2020). When LDs were inhibited with Triacsin C, the authors noted normalization in ROS, but did not comment on the restoration of cytokine output after LD inhibition. A similar observation has been made in E4 induced microglia-like (iMG) cells whose conditioned media suppressed the activity of neuronal cultures, whereas Triacsin C sufficiently reduced LD load and mitigated this suppression (Victor et al., 2022). Another recent study showed that E4/4 iMGs had significantly greater LD accumulation compared with isogenic E3/3 iMGs, and restoration of cytokines was achieved with by lowering LD load through PIK3CA inhibition (Haney et al., 2024). However, this was thought to occur through increased levels of autophagic flux and not through inhibition of droplet production.

It was our original hypothesis that the exaggerated accumulation of LDs in E4 microglia drives increased cytokine release, and thus prevention of LD formation in these cells may alleviate this pro-inflammatory phenotype. However, outside of a dampened IFN-γ response following LD inhibition, ACAT or DGAT inhibitors had minimal effect on cytokine output. There could be several possible reasons for these results. First, with no ability to form LDs in which to sequester fatty acids, the cells are vulnerable to fatty acid toxicity which could itself cause an increase in cytokine release (Lipke et al., 2022). Alternative approaches that focus on promoting LD turnover, rather than directly inhibiting their formation, could address this question and potentially provide a better approach. Second, it is possible that the increased LD accumulation and greater cytokine release in E4 microglia are simply independent phenomena – they occur in parallel, but do not share a mechanistic pathway.

Despite being largely unaffected by LD inhibition, we did observe an increase in cytokine output from E4 cells relative to E3 at baseline. However, when stimulated with LPS, E3 microglia had a more robust cytokine response, a finding that adds to a complicated literature. One possible explanation is that E4 microglia exist in a partially activated or “primed” state, which may lead to LPS tolerance, a well-characterized phenomenon in macrophages where prior exposure to inflammatory signals blunts subsequent TLR4 responses through transcriptional reprogramming (Fan and Cook, 2004; Foster et al., 2007). Additionally, APOE4 has been reported to impair TLR4 trafficking and downstream signaling in myeloid cells (Vitek et al., 2009; Liu et al., 2023), which may further limit acute responsiveness. This blunted cytokine output may also reflect metabolic constraints, as E4 LDs are depleted in fatty acid β-oxidation and oxidative phosphorylation proteins, pathways that support energy-intensive immune responses (Orihuela et al., 2016; Mills et al., 2017). However, Machlovi et al. previously showed that primary mouse microglia expressing human E4 exhibit changes in morphology and increased cytokine production associated with LD accumulation compared to E3 microglia (Machlovi et al., 2022). Additionally, several in vivo studies of E4 mice and humans show an exaggerated response to inflammation in E4 individuals (Vitek et al., 2009; Shi et al., 2017; Zhu et al., 2012; Gale et al., 2014). Conversely, Kloske et al. showed an overall reduction in inflammatory gene expression in human AD brains from E4 carriers compared to non-carriers (Kloske et al., 2021). In vitro, primary astrocytes from E4 targeted replacement mice also had a reduced cytokine response after LPS stimulation compared to E3 astrocytes (Maezawa et al., 2006), while another study noted significant sex-based cytokine secretion differences in microglia from E3 and E4 targeted replacement mice (Mhatre-Winters et al., 2022). Thus, the impact of *APOE* variants across various cell types, the role of LDs in this process, and their integration to facilitate a coordinated immune response, all remain important areas of uncertainty for the field.

Our study has several limitations. First, due to the nature of the LD enrichment, some proteins that transiently associate with LDs may have been included in the analysis. This means that proteins that are not truly LD “resident” (i.e. wholly embedded within the LD phospholipid shell) are likely included in our analyses. However, because LDs transiently interact with many other organelles (Shi et al., 2017), we believe inclusion of these interacting partners provides important insight into potential *APOE*-dependent changes in LD dynamics within the cell. In fact, if/how *APOE* may mediate organelle-organelle interactions within the cell – for example LD-mitochondria or LD-lysosomal contacts – may dictate broader functional consequences and should be a focus for future study. Second, this study only utilized female mice. This was decisions was made on the basis that women have increased susceptibility to E4 related phenotypes compared to men (Rajan et al., 2021; Yin et al., 2023), and women are disproportionately affected by AD compared to men. Future work focused E4 and LDs may revel sex dependent effects. Third, it should be noted that these results differ from recent experiments using similar methods in human induce pluripotent stem cells (hiPSC) (Stephenson et al., 2024), where inhibiting TG biosynthesis in E4 microglia blunted cytokine/chemokine release and attenuated the disease-associated transcriptional profile of these cells. Differences in ApoE and human/mouse receptor binding affinity and interactions – and their downstream effects on lipid processing (Altenburg et al., 2007; Johnson et al., 2014; Guo et al., 2025) – may explain these varying results. Fourth, we used a single, 24 h LPS challenge to capture the acute immunometabolic remodeling of the hepatic LDs. While this acute paradigm allowed us to define early lipidomic and proteomic shifts, it does not model the sustained neuroinflammation characteristic of AD. Future works employing prolonged or repeated LPS administration (or aged mice) will be needed to determine how chronic inflammation influences LD composition and function. Finally, while the current study leveraged the abundant and easily accessible LDs from the liver of mice expressing human E3 or E4, it will be important to overcome the logistical hurdles of enriching LDs from the brain and microglia in order to conduct similar profiling of human cells and tissue.

In sum, our findings demonstrate that LD composition and dynamics are modulated by APOE genotype. Integration of our novel APOE-LD proteome with previous AD and microglia datasets further suggests that many AD-implicated proteins are LD-resident, supporting a model in which LDs may serve as a central hub for immunometabolic signaling in glia. However, because lipid droplets also protect against lipotoxicity and buffer ROS, simply blocking their formation may be suboptimal. Indeed, DGAT1/ACAT1 inhibition failed to lower cytokine output in E4 microglia, indicating that future interventions may look to target LD quality and turnover rather than biogenesis alone. Possible strategies include manipulating monolayer composition (e.g. PC:PE ratios or perilipin isoform expression), enhancing β-oxidation and redirecting fatty acids into mitochondria, or accelerating lipophagy/ATGL activity to clear existing droplets. Testing these focused approaches may more effectively reverse the pro-inflammatory state of E4 microglia. Ultimately, this study and future studies modulating LD functionality or droplet proteomes holds promise in identifying future AD therapies.

## 4. Methods

### 4.1. Mouse model

Targeted replacement mice expressing human *APOE3* and *APOE4* under the endogenous *APOE* mouse promoter were used in the following experiments (Sullivan et al., 1997; Plubell et al., 2017). All mice received food and water ad libitum and were housed under a standard light/dark cycle. Procedures concerning mice were in compliance with the University of Kentucky’s Institutional Animal Care and Use Committee.

### 4.2. Lipid droplet enrichment

Twelve-month-old female mice were used to determine LD dynamics at baseline and upon inflammatory stimulation between E3 and E4 genotypes. Mice were injected with saline or lipopolysaccharide (LPS) at 5 mg/kg (*n* = 5 per group). After 24 h, mice were humanely euthanized with a lethal injection of pentobarbital. Mice were perfused with saline and 300–400 g of liver tissue was removed for lipid droplet enrichment. On ice, liver tissue was minced and bathed in homogenization buffer from Cell Biolabs Inc. (product No MET-5011). Homogenate was collected into a 2 ml vial and 600ul of density gradient separation buffer from the LD kit was gently layered on top. Samples were spun at 13,500 rpm for 3 h. A layer of lipid known as a fat-pad appeared at the top of the vial after centrifugation and this buoyant layer was enriched with lipid droplets. The top 270 μl was removed, along with the fat pad, and placed into a new tube. Two liver sections per mouse were processed this way and flash frozen in liquid nitrogen to send for proteomics and lipidomics. An additional section of liver was centrifuged and the top 270 μl was added to 1 ml of ice-cold acetone for protein precipitation and western blot analysis.

### 4.3. Proteomics analyses

Proteomics: After lipid droplet enrichment via density gradient centrifugation, the top 270ul and ‘fat pad’ containing lipid droplets was removed and placed into an Eppendorf tube. Tubes were frozen in liquid nitrogen and placed in a −80C freezer prior to shipment to BGI Global for quantitative TMT proteomics analysis using methods and normalization procedures previously described (Plubell et al., 2017). Briefly, two normalization procedures were applied for a 30-plex experiments (3 TMT experiments with 10 channels each). Within each 10-channel TMT run, the grand total reporter ion intensity for each channel was multiplied by scaling factors globally to adjust the channel intensity to the average total intensity across all ten channels in order to correct for sample loading and reaction efficiency differences. Second, common, pooled internal standards were utilized to normalize reporter ion intensities between the three TMT experiments.

Proteomics analysis detected 6192 proteins. Proteins were accepted if they contained more than two unique peptides and were within the false discovery rate of 5 %. A final 4267 proteins were included and analyzed. These final proteins are considered as both LD-resident proteins and LD- interacting proteins. To determine which proteins may be true LD resident proteins, the accepted 4267 were referenced against 6 known lipid droplet proteomes from liver cells and mouse and rat liver tissue (Liu et al., 2023; Olzmann & Carvalho, 2019; Mejhert et al., 2020; Khan et al., 2015; Krahmer et al., 2018). Upon cross-referencing our database with the six others, 2099 overlapping proteins were identified and considered as LD-resident proteins. Abundance averages were calculated within groups and multiple group *t*-tests were performed on the entire dataset. Significantly differentiated proteins (−log10(*p*-value) > |1.3|) were plugged into the open-source gene ontology analysis site, Enrichr, to understand protein interactions within biological processes. Volcano plots were made for each condition in the entire data set utilizing raw *p*-values. Our full database was also compared against proteomes from papers investigating differences in the brain, Alzheimer’s disease, and microglia (Krasemann et al., 2017; Casey et al., 2021; Roberts et al., 2021; Johnson et al., 2020; Johnson et al., 2022). We analyzed overlapping proteins from our database with these papers and further investigated their contribution to our hypothesis.

### 4.4. Lipidomics analyses

After LD enrichment via density gradient centrifugation, the top 270 μl and fat pad containing LDs was removed and placed into an Eppendorf tube. Tubes were frozen in liquid nitrogen and placed in a −80 °C freezer. Samples were packaged and sent to BGI global. Nontargeted lipidomics analysis was performed using LC-MS/MS. High resolution mass spectrometer Q Exactive (Thermo Fisher Scientific, USA) was used for data acquisition in positive-ion and negative-ion mode respectively to improve the lipid coverage. The data were processed by LipidSearch 4.1 and BGI’s statistical software package, metaX. Lipidomic analysis revealed 321 unique lipid metabolites. An outlier analysis was performed in Prism Graphpad using the GRUBS method with an alpha = 0.0001 (repeated untill 0 outliers remained) which revealed 28 statistical outliers among the 6420 total values (321 metabolites × 20 samples; 0.4 % outliers). Metabolite abundance averages were calculated within groups and multiple group *t*-tests were performed on the entire data set followed by post-hoc multiple comparisons correction for false discovery rate (FDR) using Benjamini-Hochberg (BH) adjustment.

### 4.5. Oil Red O staining and analysis

Liver sections from mice treated with LPS or saline were stained with Oil Red O and Hematoxylin to quantify lipid droplet (LD) accumulation. Sections were imaged on the Zeiss Axio Scan Z1, and three areas per sample were selected for droplet analysis. Lipid droplet area was quantified using an automated algorithm that separated colors to highlight the red-stained droplets. For image processing, a Gaussian filter was applied using Python’s OpenCV module, followed by color detection in the RGB space to segment the lipid droplets. The non-black pixels were counted to estimate droplet area. Droplet size was calculated using ImageJ, based on circularity measurements and area under the curve analysis.

### 4.6. WGCNA

Weighted gene co-expression network analysis (WGCNA) (v1.70–3) was used to identify gene modules and build unsigned co-expression networks, including both negative and positive correlations. Briefly, WGCNA constructs a gene–gene adjacency matrix (using Pearson correlation), then raised it to a soft-thresholding power β to approximate scale-free topology and then uses hierarchical clustering to group genes into modules of closely co-expressed features. We first selected the top 3000 most variable genes for network construction. Soft power β = 6 was chosen via the pickSoftThreshold function, at which the scale-free fit index (Olzmann and Carvalho, 2019) exceeded 0.85, while preserving adequate mean connectivity. Next, TOMsimilarityFromExpr (power = 6, networkType = “signed”) was used to compute the Topological Overlap Matrix, and a dissimilarity matrix (1–TOM) was clustered with flashClust (v1.01). Modules were then defined by cutreeDynamic (deepSplit = 3, minModuleSize = 30). To assess module quality, we calculated for each module the mean absolute module membership (|kME|) and the proportion of “hub” genes (|kME| > 0.8). Across all modules, mean |kME| values ranged from 0.80 to 0.83 and 56–74 % of genes per module were classified as hubs, demonstrating high intramodular connectivity and module cohesion. Finally, module-trait relationships were quantified via Pearson correlations between module eigengenes and sample traits (APOE genotype and treatment), and the gene–gene network for all modules was exported for visualization in Cytoscape (v3.8.2). The top 10 hub proteins displayed in each module network (Fig. 4c–d) were selected based on highest module membership (kME), indicating strongest intramodular connectivity, and all proteins assigned to highlighted modules were used to perform gene ontology using web based enrichR.

### 4.7. Western blotting

Protein from the lipid droplet enriched fraction was precipitated with acetone and resuspended in RIPA with protease inhibitor. The sample was diluted with 10 μl MilliQ water, and then further diluted at a 1:1 ratio with 2× Laemmli Sample Buffer (Bio-Rad Laboratories, Hercules, CA, USA). The samples were heated at 96.5° Celsius for 10 min, after which they were chilled on ice for 5 min before loading into the gel. 20 μl of the protein sample was loaded on 4–20 % Criterion TGX Gels (Bio-Rad Laboratories, Hercules, CA, USA). Gels were transferred onto 0.2 μm nitrocellulose membrane (Bio-Rad Laboratories, Hercules, CA, USA) using the Trans-Blot Turbo Transfer System (Bio-Rad Laboratories, Hercules, CA, USA). After transfer, membranes were blocked for 30 min in 1 % casein solution while gently rocking back and forth using the VWR Analog Rocker (Avantor, Radnor, PA, USA). The membranes were incubated overnight at 4° Celsius in 1:1000 PLIN-2 primary antibody solution (Novus Biologicals, Centennial, CO, USA). After incubation, the membranes were washed with PBS-T (0.05 % Tween-20), three times for five minutes each wash. The membranes were then incubated for one hour at room temperature, while protected from light, in 1:5000 Goat α-rabbit IR 700 secondary antibody solution. (Bio-Rad Laboratories, Hercules, CA, USA). They were then washed with PBS-T, three times for five minutes each, and then with PBS two times for five minutes each. Membranes were imaged using a ChemiDoc XRS Imaging System (Bio-Rad Laboratories, Hercules, CA, USA).

### 4.8. Cell culture

Human *APOE* expressing mice were bred and pups were taken at postnatal day P1–3 to obtain primary mouse microglia for experiments. Littermates were pooled together to increase cell counts, but pups from the same litter were not genotyped for sex chromosomes. During dissection, forebrains were removed from mice and placed in a dissection buffer consisting of ice-cold Hanks Balanced Salt Solution (HBSS #). Under the dissection scope, meninges were carefully removed and the brain was placed in DMEM F12 media (10 % FBS and 1 % pen/strep). Pooled brains were cut into small pieces and digested with trypsin at 37C for 25 min, agitating every five minutes. Trypsin was neutralized with an equal volume of DMEM media and brains were centrifuged for 5 min at 400 ×*g* prior to washing with HBSS. Brain homogenate was resuspended in DMEM, triturated, and ran through a 70 μm filter. Mixed glial cells from this preparation were split into T75 flasks (1 flask per brain). Dead cells and debris were washed after day 1 in culture and media was switched to a supplemented DMEM: F12 media on day 7. After two weeks, microglia begin to emerge from the glial cells. Flasks were placed on an orbital shaker for two hours and media was collected and centrifuged to pellet the microglia. Cells were resuspended in DMEM, counted, and plated for various experiments.

### 4.9. Lipid droplet imaging

For lipid droplet imaging experiments, cells were seeded at 60,000 cells per well on a poly-L-lysine coated 12 mm glass coverslip. Cells were treated with 250uM oleic acid conjugated with BSA, 10 ng lipopolysaccharide (LPS), necroptotic Neuro-2 A cells (nN2A), N2A plus LPS, oleic acid plus LPS, or an untreated control. After 24 h, media was collected for cytokine analysis and cells were fixed in 4 % paraformaldehyde. Cells were stained with BODIPY, and coverslips were mounted with a DAPI nuclear staining mounting media. N2A cells were split into a few dozen T-75 flasks with non-filter cap lids. Once cells grew to confluency, the caps were tightened to induce hypoxia and cell death. Cells began to detach from the flasks and all apoptotic cells and media were collected, split into 8 × 10^6^ cells/ml aliquots and frozen for later use. Microglia were treated at a 1:10 ratio of microglia to nN2A cells during experiments.

Imaging took place on a Nikon A1R confocal microscope. 405 nm wavelength was used to acquire microglial nuclei stained with DAPI and 488 nm wavelength was used to acquire neutral lipid stained with BODIPY. Color threshold analysis using ImageJ was used to select high intensity regions of neutral lipid, indicating the presence of lipid droplets. Lipid droplets were then quantified by measuring the threshold area.

### 4.10. Cytokine analysis

Primary mouse microglia from E3 and E4 targeted replacement mice were plated in 96-well plates and allowed to adhere. For a 24-h treatment, cells were treated with either a DMSO control, DGAT inhibitor (10 μM A922500), ACAT inhibitor (10 μM Avasimibe), or both inhibitors. Six conditions were tested: control, 250 μM OA, 10 ng LPS, 1:10 ratio of nN2A cells, OA + LPS, and nN2A + LPS. Cytokines were measured using a custom V-plex assay from MesoScale Discoveries with probes for IL-1ß, INF-y, TNF, IL-10, and IL-6.

## Supplementary Material

1

2

3

Appendix A. Supplementary data

Supplementary data to this article can be found online at https://doi.org/10.1016/j.nbd.2025.106983.

## Data Availability

Data has been provided in the supplemental table.
