# Supplementary material for: APOE4 reshapes the lipid droplet proteome and modulates microglial inflammatory responses
Source: Neurobiol Dis. Author manuscript; Available in PMC 2025 Aug 1. (PMC12187248; doi:10.1016/j.nbd.2025.106983)
Supplement: 1 [file NIHMS2090365-supplement-1.docx]

**Supplemental Figures**

**
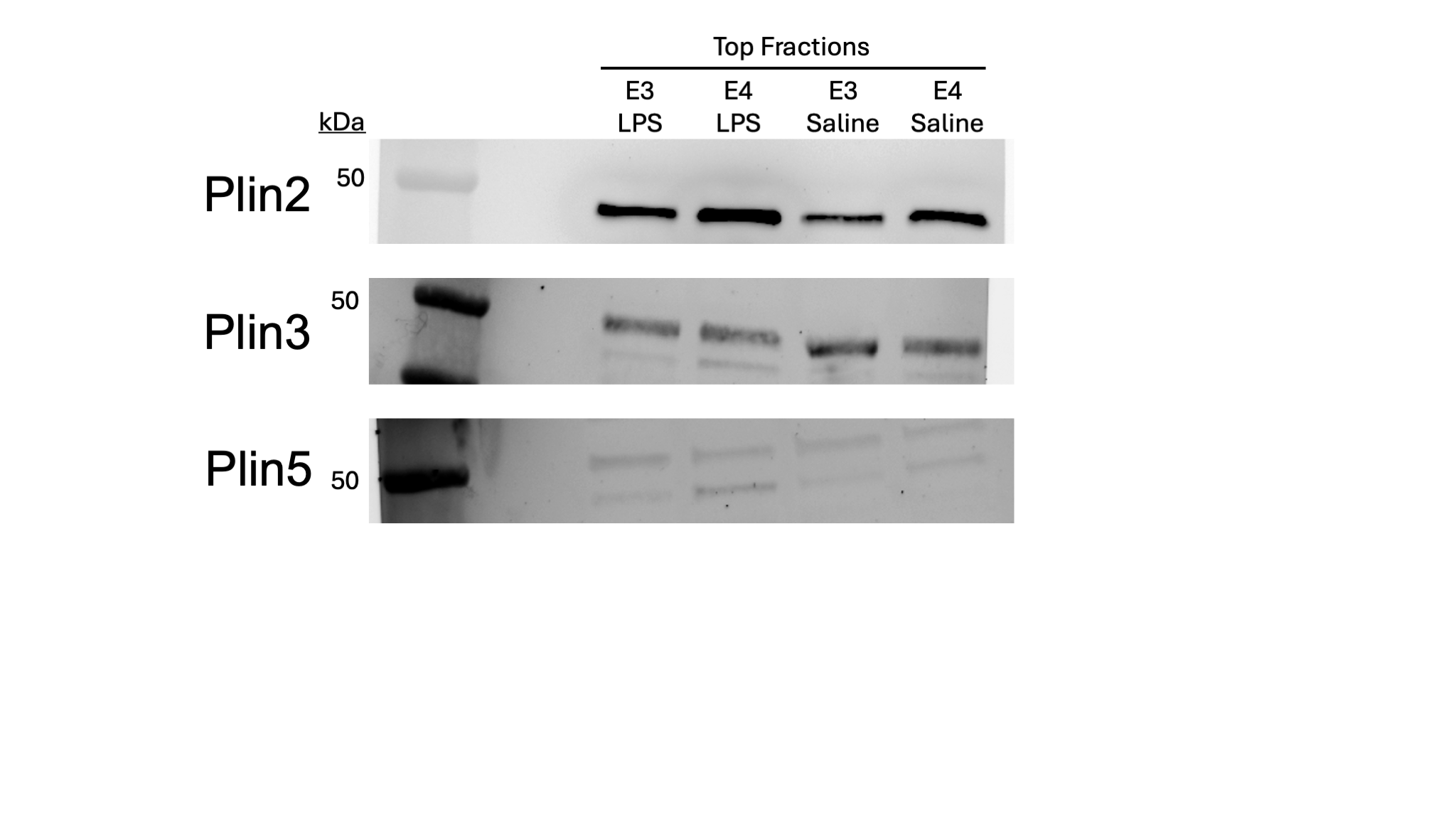
**

**Supplementary Figure 1. Western blot analysis of perilipin proteins in lipid droplet-enriched top fractions from E3 and E4 mouse livers.** Lipid droplet-enriched top fractions were isolated from livers of APOE3 and APOE4 targeted replacement mice treated with saline or LPS. Western blots were performed to detect Plin2, Plin3, and Plin5. Plin2 was highly enriched in LD fractions and increased with LPS treatment across genotypes. Plin3 and Plin5 appeared as multiple bands, possibly reflecting post-translational modifications such as phosphorylation.


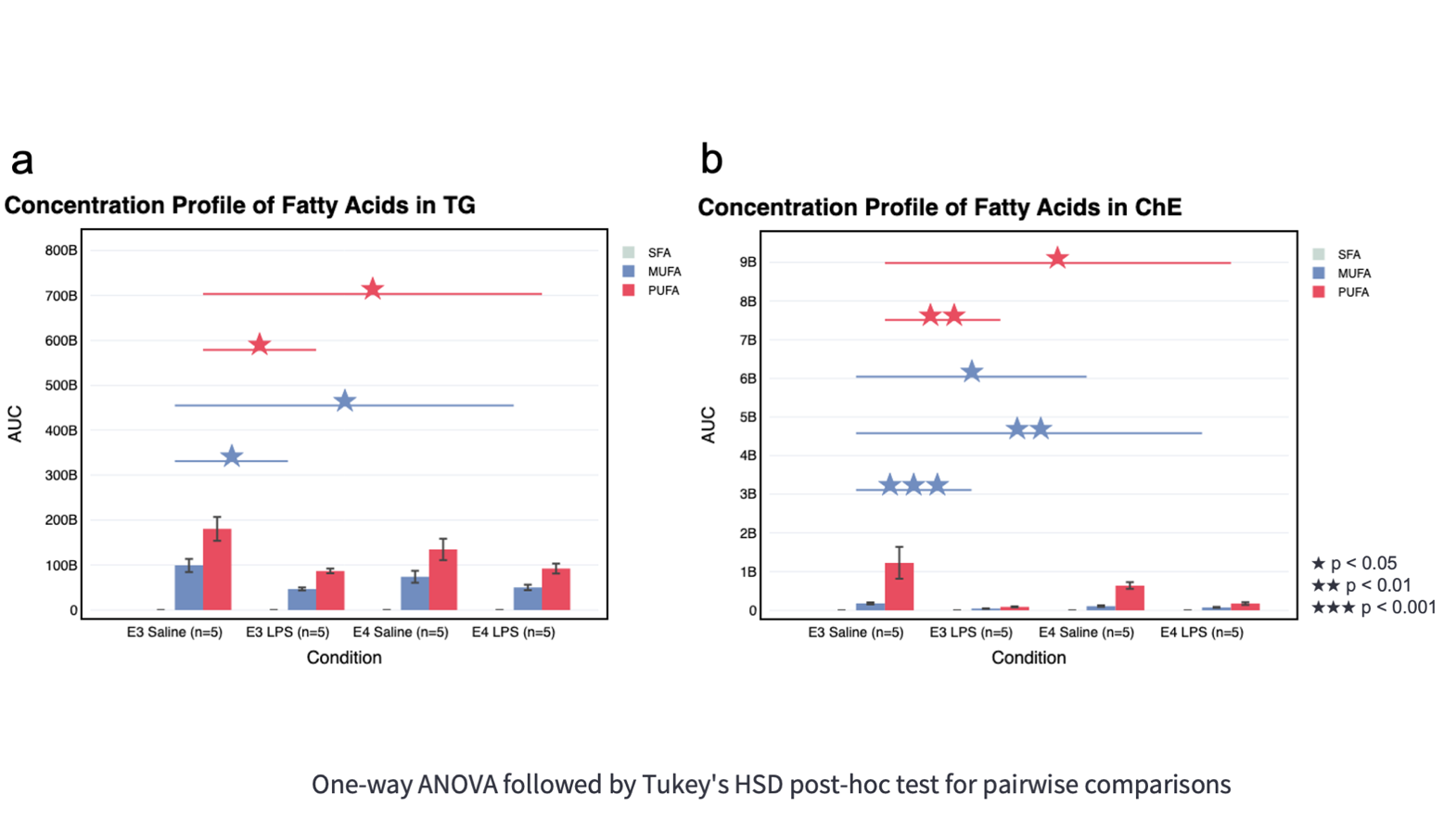


**Supplementary Figure 2. Saturation profile of triglyceride and cholesterol ester species within lipid droplets.** a) Concentration of saturated (SFA), monounsaturated (MUFA), and polyunsaturated fatty acids (PUFA) in triglycerides (TG) isolated from lipid droplets (LDs) of E3 and E4 targeted replacement mice treated with saline or LPS. b) Concentration of SFA, MUFA, and PUFA in cholesterol esters (ChE) from the same LD fractions. Statistical comparisons were performed using one-way ANOVA with Tukey’s post hoc test for multiple comparisons. **Abbreviations:** PUFA, polyunsaturated fatty acid; MUFA, monounsaturated fatty acid; SFA, saturated fatty acid; TG, triglyceride; ChE, cholesterol ester; LD, lipid droplet; LPS, lipopolysaccharide. Plots were generated using lipidcruncher.org. https://www.biorxiv.org/content/10.1101/2025.04.28.650893v1


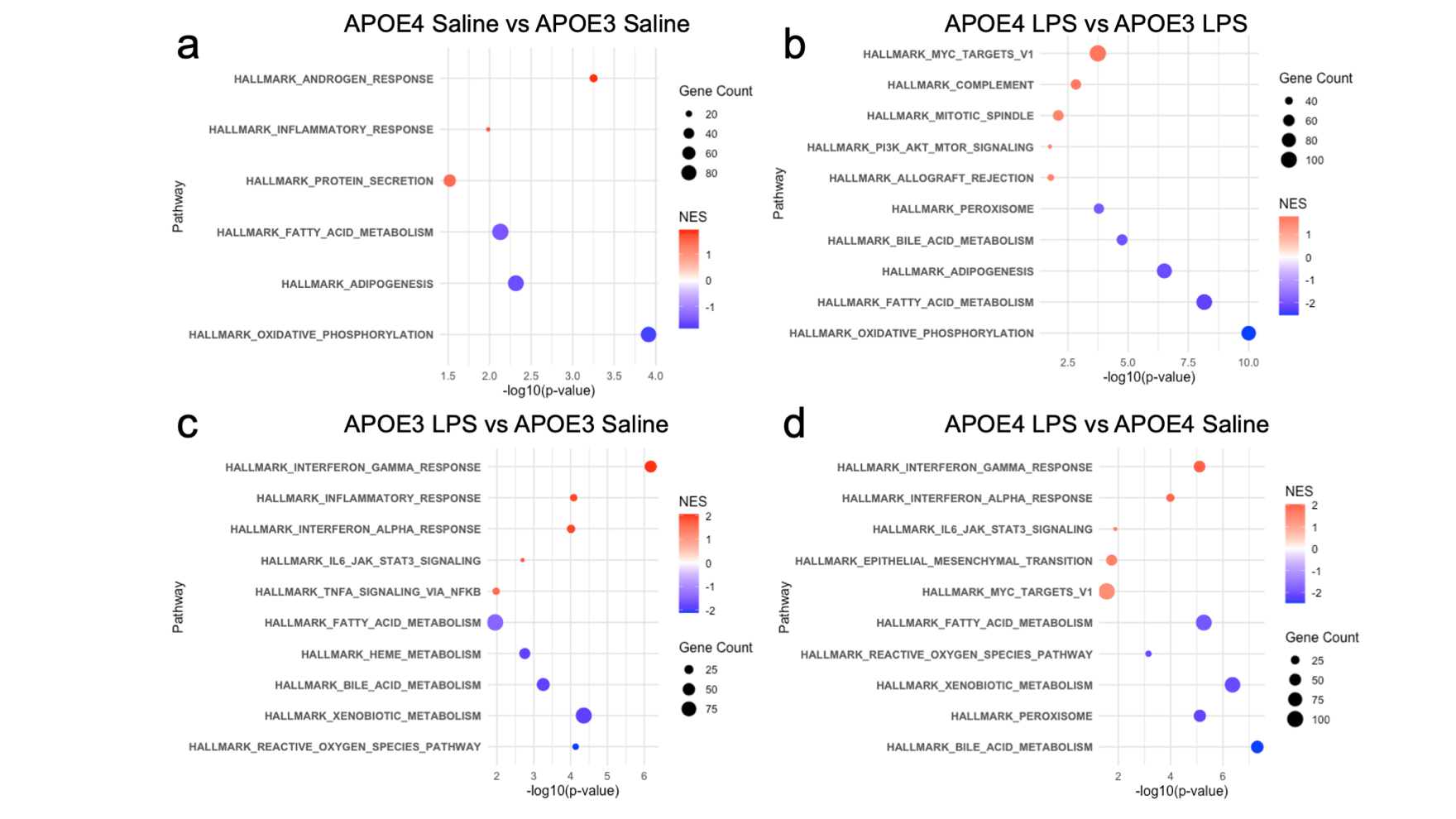
**Supplementary Figure 3. Hallmark pathway enrichment reveals APOE- and treatment-specific differences in the lipid droplet proteome.** Gene set enrichment analysis (GSEA) was performed using the MSigDB mouse Hallmark pathway database to identify pathways enriched across genotype and treatment conditions. a–d) Dot plots display significantly enriched Hallmark pathways in the lipid droplet (LD) proteome across each comparison. Pathways enriched in the first group listed are shown in red (positive normalized enrichment score [NES]), and those enriched in the second group listed are shown in blue (negative NES). a) APOE4 vs. APOE3 LDs under saline conditions. b) APOE4 vs. APOE3 LDs following LPS treatment. c) LPS- vs. saline-treated APOE3 LDs. d) LPS- vs. saline-treated APOE4 LDs. Pathways are ranked by NES and plotted by –log10(p-value). **Abbreviations:** GSEA, gene set enrichment analysis; NES, normalized enrichment score; LPS, lipopolysaccharide.


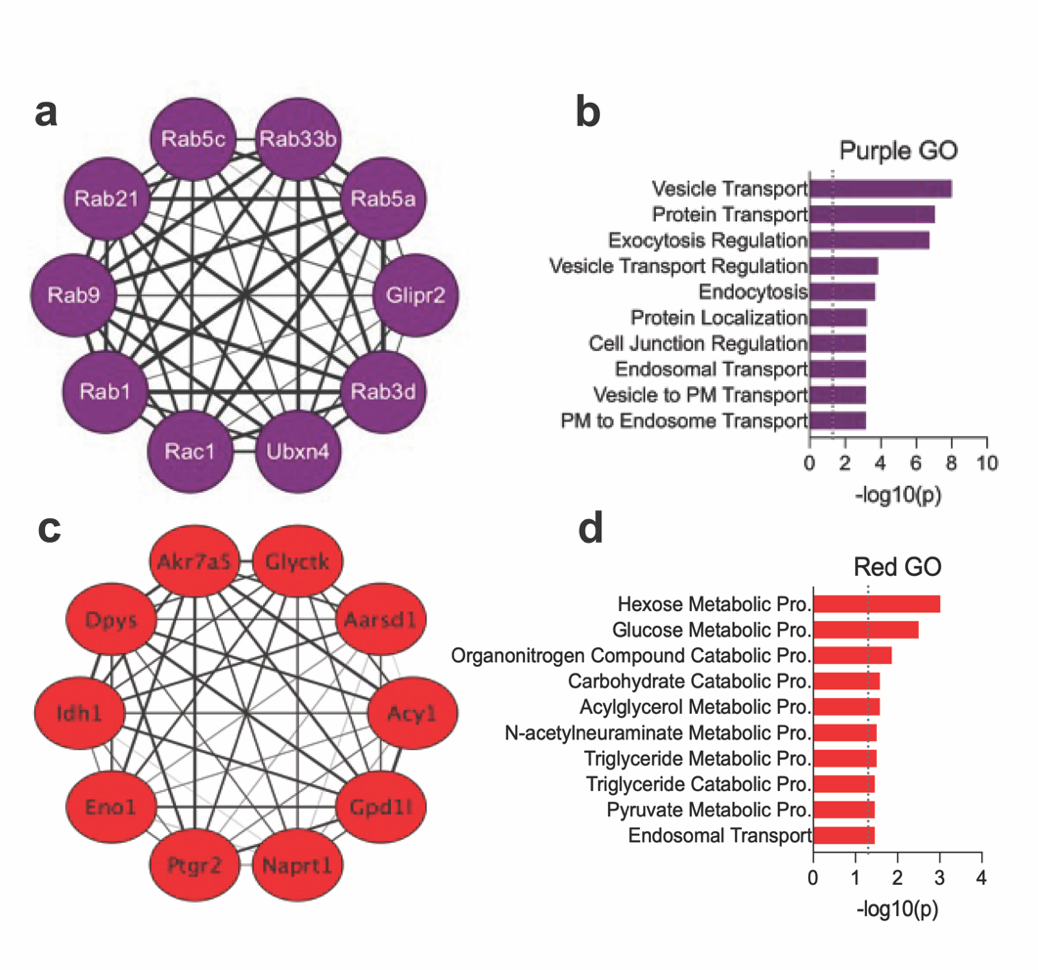


**Supplementary Figure 4. The purple and red WGCNA modules are enriched for vesicle trafficking and carbohydrate metabolism, respectively.** a–b) The purple module, which was significantly upregulated in both E3 and E4 microglia following LPS treatment, is enriched for proteins involved in vesicle transport and protein trafficking. a) Network diagram showing the top 10 hub proteins (highest kME) in the purple module. b) Gene ontology (GO) enrichment of all proteins in the purple module. c–d) The red module was downregulated in both genotypes following LPS and is enriched for carbohydrate metabolism and triglyceride biosynthetic processes. c) Network diagram showing the top 10 hub proteins in the red module d) GO enrichment of all proteins in the red module.


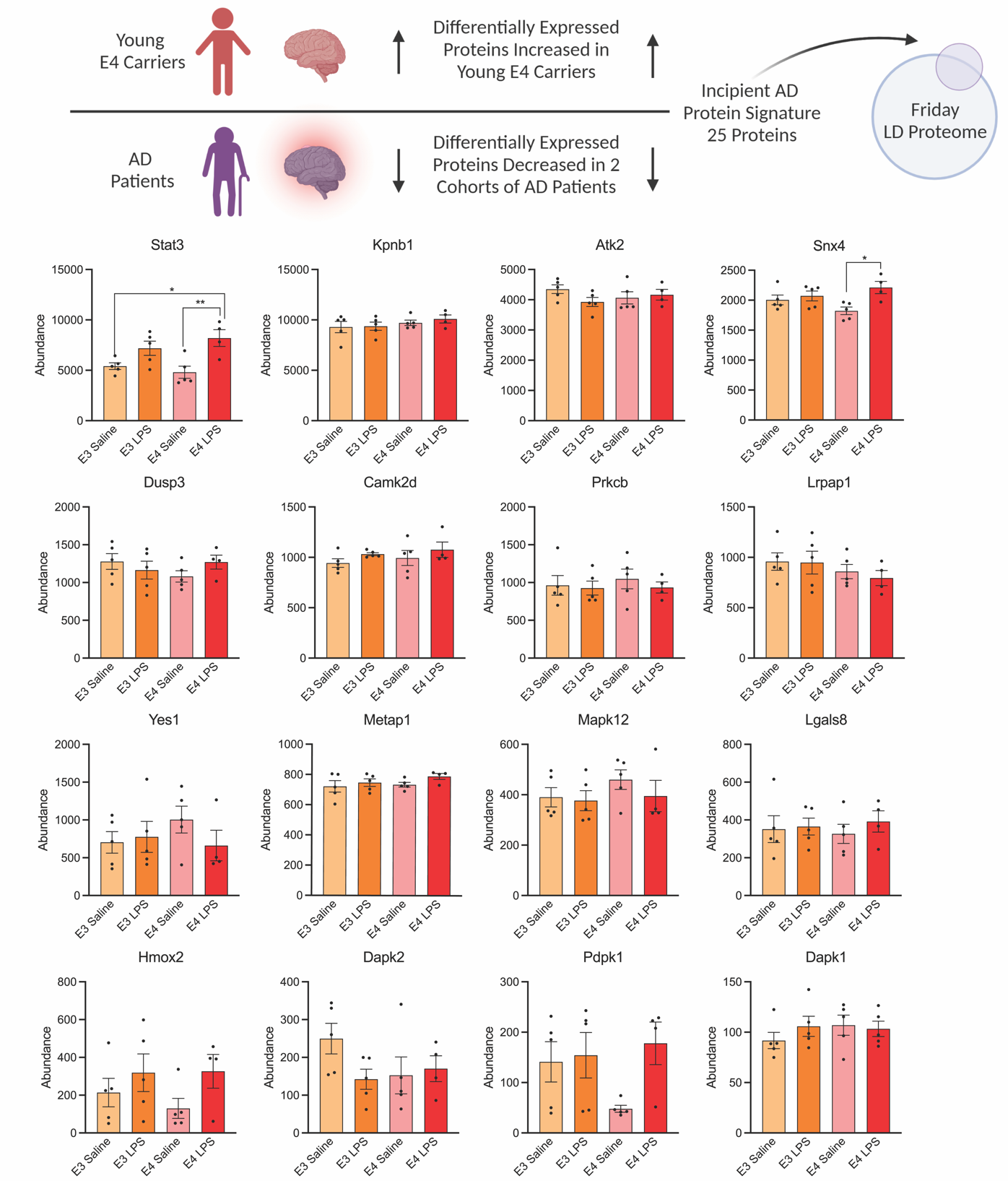


**Supplement Figure 5.** This “incipient AD” (iAD) signature showed 60% overlap with the APOE LD proteome from the current study. Shown are the individual graphs for each of the overlapping proteins. *p<0.05, **p<0.005, two-way ANOVA analyses


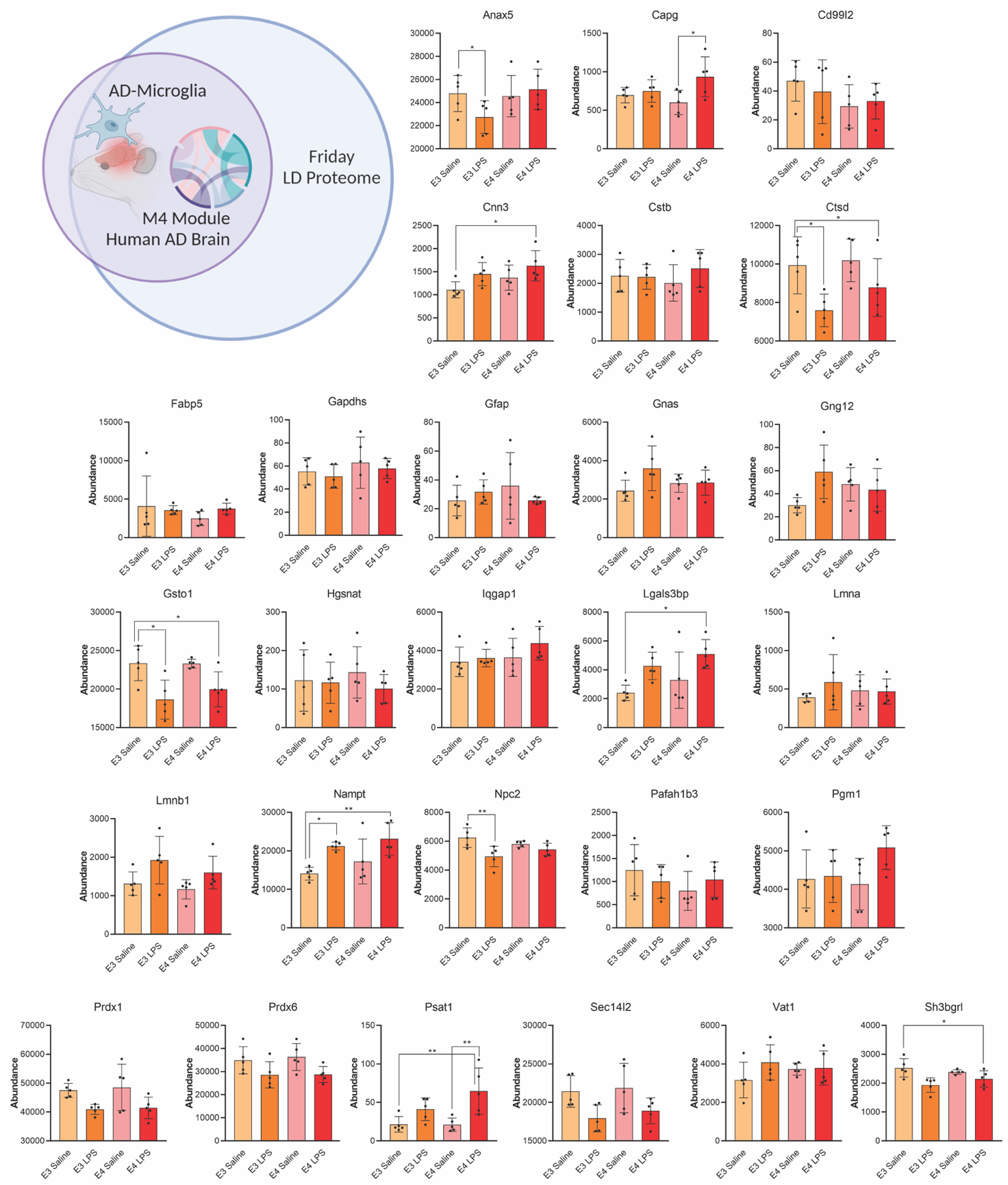


**Supplement Figure 6.** A large proteomic study by Johnson et al. revealed a glial metabolism module (“M4”) which was differentially expressed in AD brains as well as in microglia from a mouse model of AD. This AD-glial-metabolism signature has 90% overlap with our LD proteome. Shown are the individual graphs for each of the overlapping proteins. *p<0.05, **p<0.005, two-way ANOVA analyses.


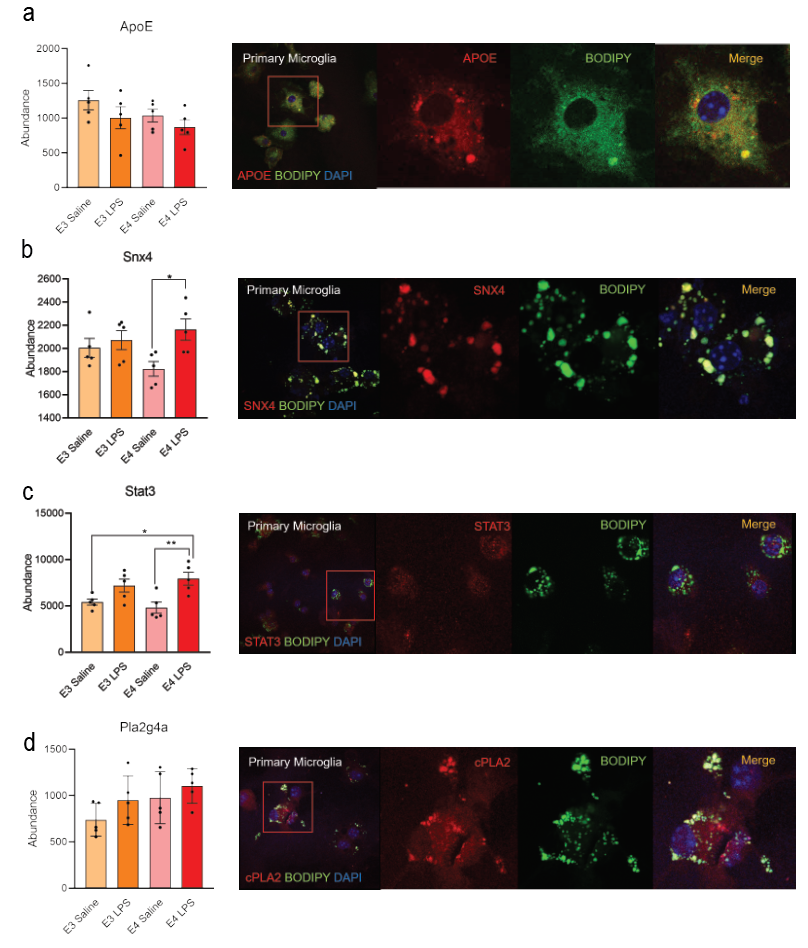


**Supplementary Figure 7. Localization of lipid droplet-associated proteins in primary microglia.** a–d) Bar graphs show protein abundance levels across conditions (left), and immunofluorescence images show subcellular localization of each protein (right). BODIPY 493/503 was used to label lipid droplets. a) APOE colocalizes with BODIPY in primary microglia, confirming its association with lipid droplet surfaces. b) SNX4, an “incipient AD” (i-AD) protein implicated in endosomal trafficking and AD pathology, also localizes to the droplet surface. c) STAT3, another i-AD protein involved in immune signaling, is present in the cell but does not colocalize with lipid droplets. d) PLA2G4A (also known as cPLA2), a phospholipase linked to the eicosanoid pathway, is detected on lipid droplets in primary microglia, consistent with its known presence in liver LDs.


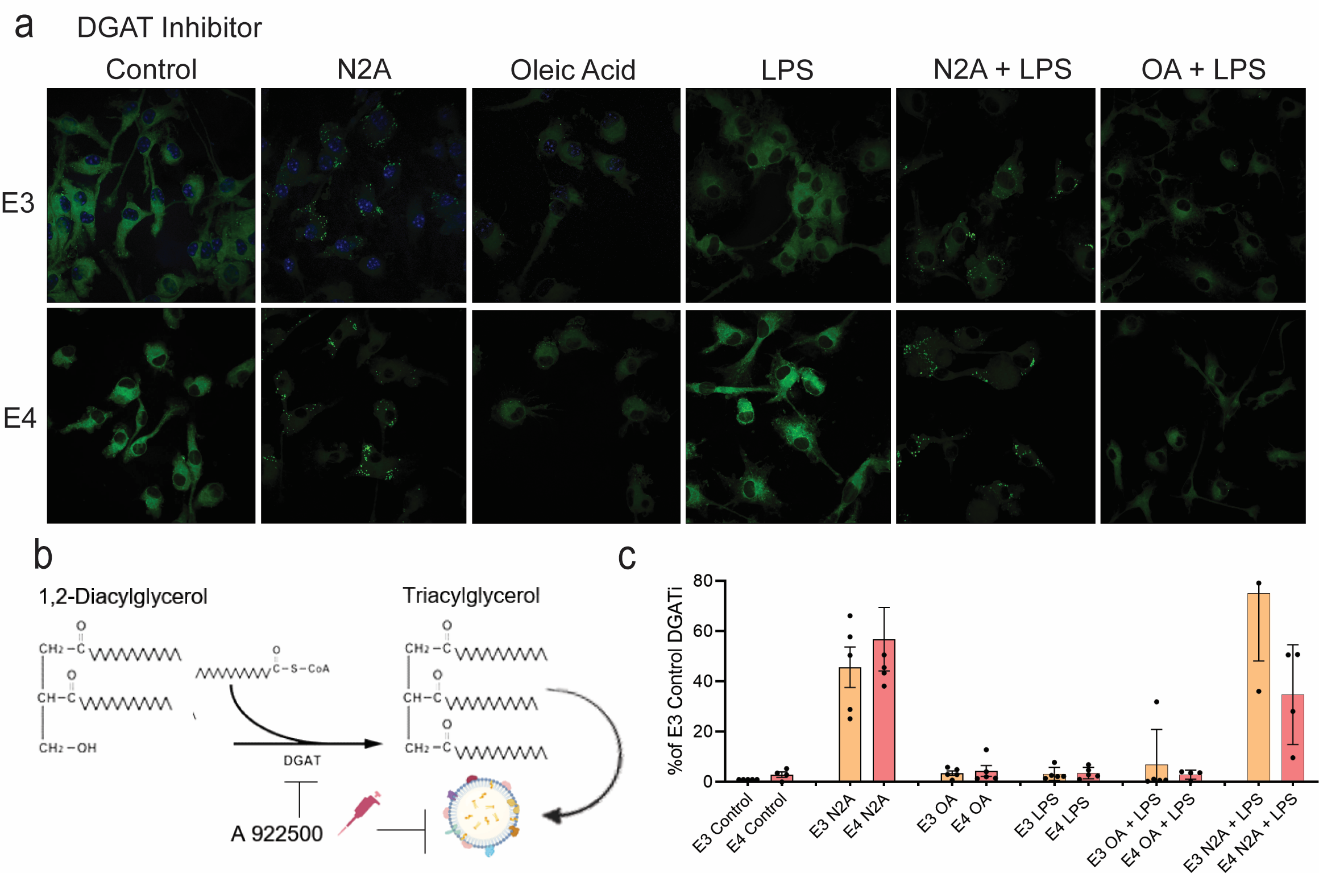


**Supplementary Figure 8. DGAT1 inhibition suppresses lipid droplet formation in microglia except under necrotic neuron exposure.** a) BODIPY staining of primary microglia treated with the DGAT1 inhibitor A922500 shows reduced lipid droplet (LD) accumulation across most stimuli, including oleic acid and LPS, but LDs persist in cells treated with necrotic N2A cells (nN2A). b) Schematic of the DGAT pathway: DGAT1 catalyzes the final step in triacylglycerol (TAG) synthesis from diacylglycerol (DAG), driving TAG storage into LDs. A922500 blocks this step by inhibiting DGAT1. c) Quantification of BODIPY-positive area per cell confirms DGAT1 inhibition reduces LD formation in all groups except those treated with nN2A. This suggests that necrotic neurons may activate a compensatory lipid storage pathway, likely via increased ACAT activity.


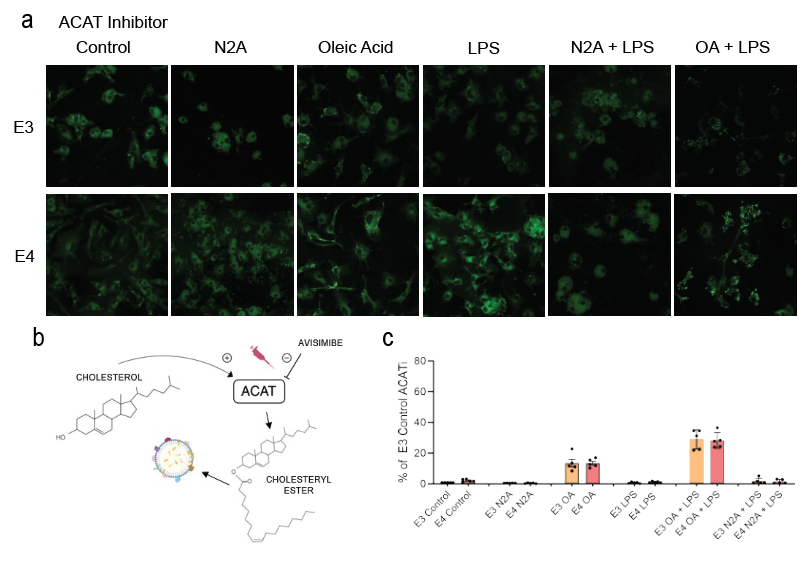


**Supplementary Figure 9. ACAT inhibition suppresses lipid droplet formation except under oleic acid treatment**a) BODIPY staining of primary microglia treated with the ACAT inhibitor Avasimibe reveals reduced lipid droplet (LD) accumulation under all conditions except oleic acid (OA) treatment, where LDs persist. b) ACAT catalyzes the esterification of cholesterol into cholesterol esters (ChE), which are stored in LDs. Avasimibe inhibits this process and reduces ChE-based LD biogenesis. c) Quantification of BODIPY-positive area per cell confirms that Avasimibe suppresses LD formation equally in both E3 and E4 microglia across most conditions. However, LD accumulation persists in OA-treated groups, likely due to continued triacylglycerol (TAG) synthesis via the DGAT pathway, which is not blocked by ACAT inhibition.


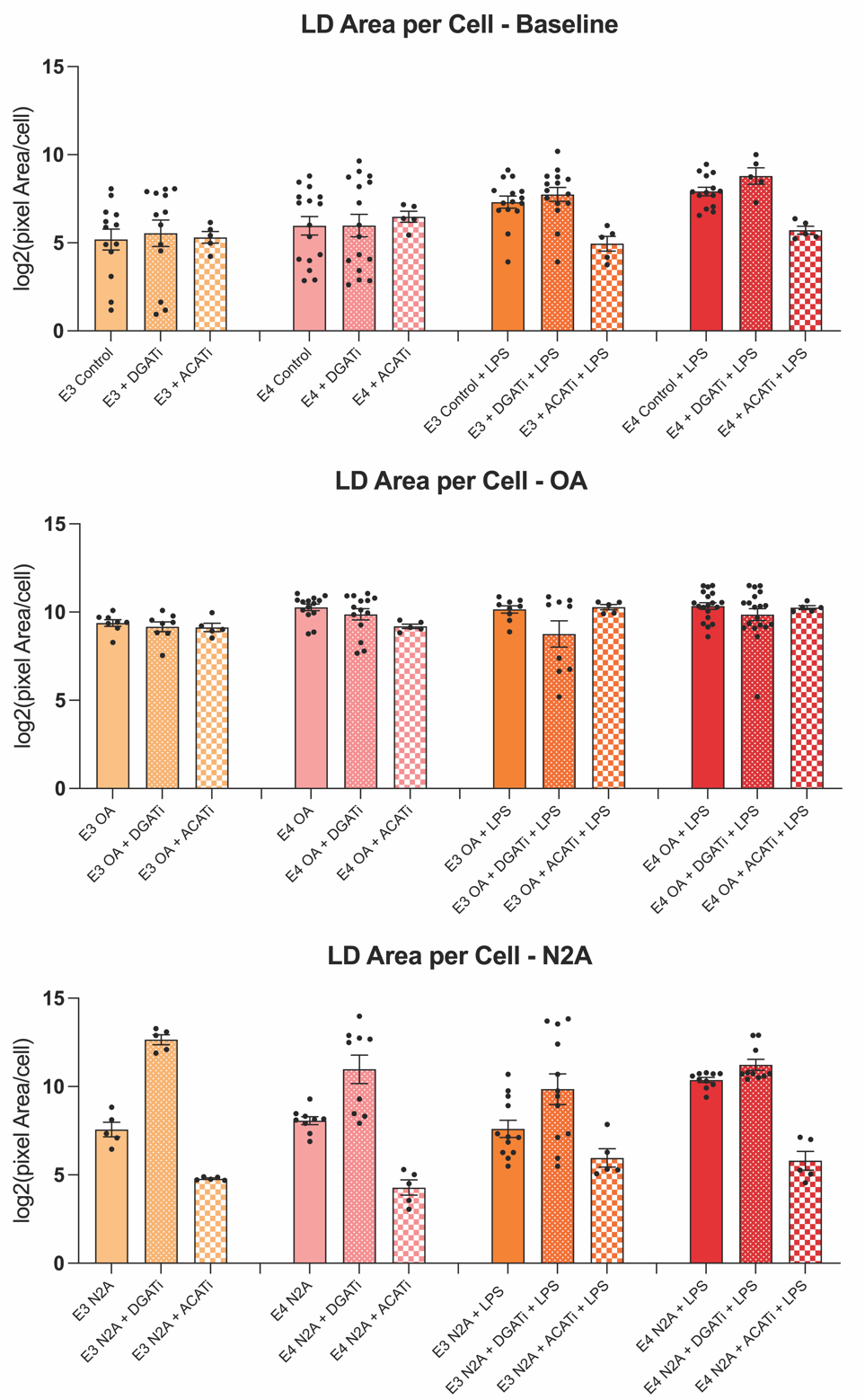


**Supplement Figure 10. Quantification of lipid droplet accumulation across microglial treatments reveals genotype- and stimulus-specific effects of DGAT and ACAT inhibition.** BODIPY-labeled lipid droplet (LD) area per cell was quantified in E3 and E4 primary microglia treated with various stimuli and inhibitors. Each bar represents the mean ± SEM of log2-transformed LD area per cell. **Top panel:** Baseline conditions (control, nN2A, or OA) without LPS. **Middle panel:** OA-treated conditions with or without LPS, DGAT inhibition (A922500), or ACAT inhibition (Avasimibe). **Bottom panel:** nN2A-treated conditions with or without LPS, DGAT inhibition, or ACAT inhibition. LD accumulation was generally higher in E4 microglia, particularly after exposure to nN2A. Both DGAT and ACAT inhibitors reduced LD area in most conditions, though LDs persisted in OA-treated groups following ACAT inhibition and in nN2A-treated groups following DGAT inhibition—suggesting substrate-specific bypass of lipid storage pathways.


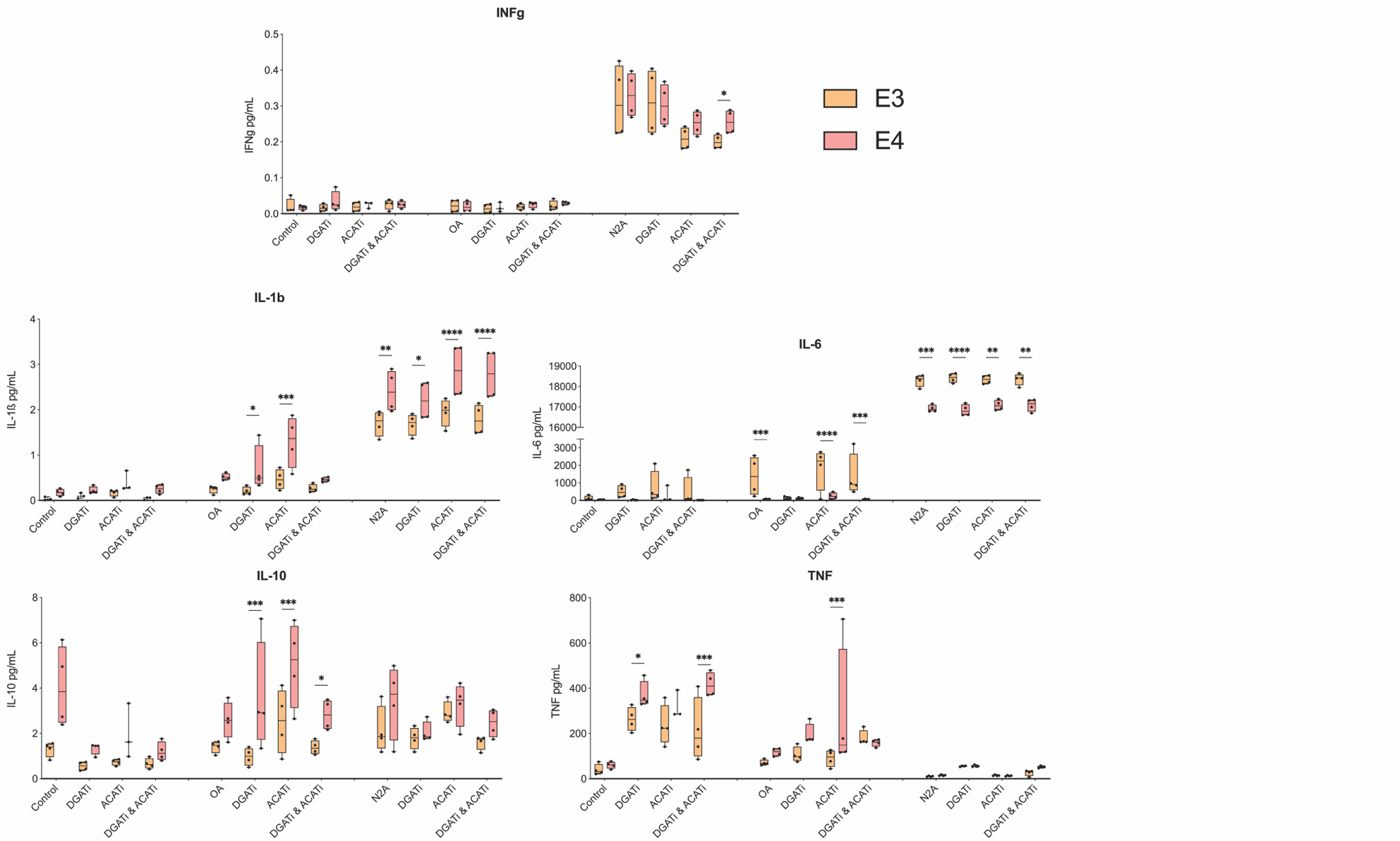


**Supplementary Figure 11. Cytokine secretion from E3 and E4 microglia following treatment with lipid-loading stimuli and lipid droplet inhibitors.** Concentrations of IFNγ, IL-1β, IL-6, IL-10, and TNF were measured in the media of primary E3 and E4 microglia following treatment with necrotic N2A cells (nN2A) or oleic acid (OA), in the presence or absence of DGAT inhibitor (A922500), ACAT inhibitor (Avasimibe), or both. E4 microglia secreted higher levels of pro-inflammatory cytokines (IL-1β, IL-6, TNF) across most conditions. Inhibition of lipid droplet formation variably altered cytokine release, with combined DGAT and ACAT inhibition often producing the strongest suppression. Data are presented as box-and-whisker plots; *p < 0.05, **p < 0.01, ***p < 0.001, ****p < 0.0001 by two-way ANOVA with multiple comparisons.


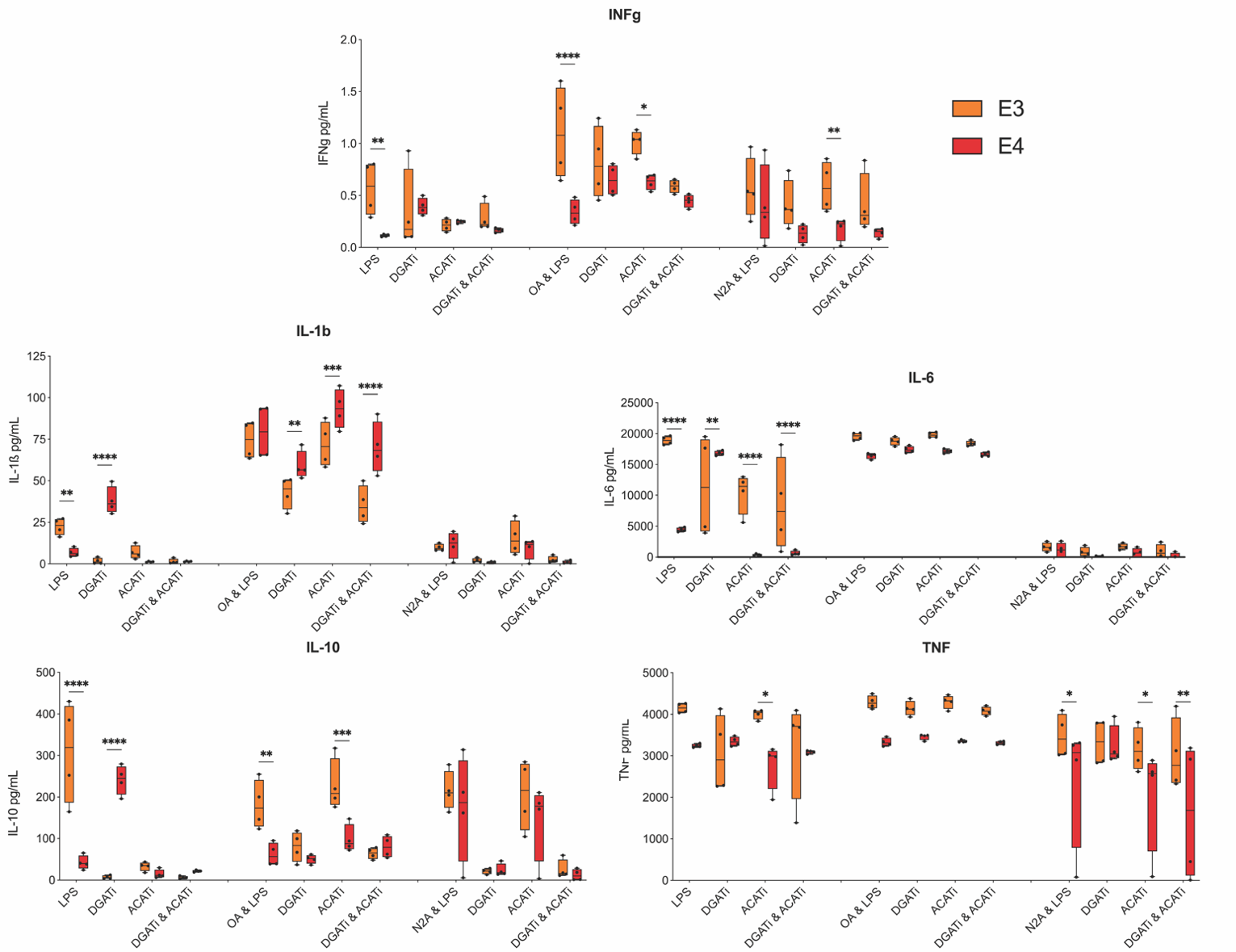


**Supplementary Figure 12. Lipid droplet inhibition modulates cytokine secretion in LPS-treated E3 and E4 microglia.**
Concentrations of IFNγ, IL-1β, IL-6, IL-10, and TNF were measured in the media of E3 and E4 primary microglia following LPS stimulation. Cells were pretreated with vehicle, DGAT inhibitor (A922500), ACAT inhibitor (Avasimibe), or both, in combination with either oleic acid (OA) or necrotic N2A cells (nN2A) as lipid-loading stimuli. Cytokine profiles varied by genotype and treatment, with E3 microglia generally producing higher cytokine levels after LPS, particularly under OA + LPS conditions. Lipid droplet inhibition blunted cytokine release to varying degrees, with the combination of DGATi and ACATi often yielding the strongest suppression. Data are shown as box-and-whisker plots. *p < 0.05, **p < 0.005, ***p < 0.001, ****p < 0.0001 by two-way ANOVA with multiple comparisons.
