# Supplementary material for: APOE4 reshapes the lipid droplet proteome and modulates microglial inflammatory responses
Source: Neurobiol Dis. Author manuscript; Available in PMC 2025 Aug 1. (PMC12187248; doi:10.1016/j.nbd.2025.106983)
Supplement: 2 [file NIHMS2090365-supplement-2.docx]

**Supplement Methods:**

**Oil Red O staining and analysis**

Liver sections from mice treated with LPS or saline were stained with Oil Red O and Hematoxylin to quantify LD accumulation. Dissected liver specimens were fixed in 4% PFA for 24 hours then sunk in 30% sucrose + 0.05% sodium azide before being fixed in O.C.T. Free-floating sections were cut at 10μM and washed 3x for 5 minutes with PBS prior to staining. Sections were placed in 100% propylene glycol for 5 minutes then incubated in pre-heated Oil Red O (Abcam #150678) for 6 minutes at 60°C. After staining, sections were placed in 85% propylene glycol for 1 minute, rinsed with 2 changes of distilled water, and counterstained with Hematoxylin. Sections were mounted with ProLong Gold upon air drying. Liver sections were imaged on the Zeiss Axio Scan Z1 and 3 areas from each sample were randomly selected for droplet analysis. Total lipid droplet area was assessed using an automated algorithm which removed white (blank) background from the images and separated the colors to highlight the red-stained droplets. Individual droplet area was evaluated using ImageJ based on circularity of the droplets.

ORO images were loaded in a BGR format using the OpenCV module in Python. A Gaussian filter with a kernel value of 5 was applied to the images in order to reduce the noise of the pixels. The now blue lipid droplets were thresholded by performing color detection of the range of red (corresponding to the other tissue), using the cv2.inRange function and defining the boundaries in the RGB color space. The lower limit of the color was set as [60, 20, 2], and the upper limit, as [126, 255, 255]. A binary mask was then imposed on top of the original image, producing a new one where the values of the pixels within the defined color range become 0 (black). Upon counting the non-black pixels, that number was then multiplied by 100 and divided by the total amount of pixels conforming the image. The segmented images were saved into a database.

For individual lipid droplet area calculation, the segmented images were imported into ImageJ and subsequently turned into 8-bit grayscale images using the command Image>Type>8 bit (black and white). A new mask was imposed on top of the image using the command Image>Adjust>Threshold, setting the lower limit as 0 and the upper limit as 20 to re-cover the black pixels in the image. Upon inverting the colors of the image using the command Edit>Invert, the droplets were quantified and measured using the command Analyze>Analyze particles; the pixel size was set to 0-100 and circularity was set to 0 (1 being a perfect circle). Configurations were set to display the resulting outlines as new images. Perimeter and area measurements were selected and found under the menu Analyze>Set measurements. Individual droplet count and area was used to create a LD size histogram. Total LD area per image was calculated based on an area under curve analysis of this generated histogram.

**Western Blots**

Protein from the lipid droplet enriched fraction was precipitated with acetone and resuspended in RIPA with protease inhibitor. The sample was diluted with 10 ml MilliQ water, and then further diluted at a 1:1 ratio with 2x Laemmli Sample Buffer (Bio-Rad Laboratories, Hercules, CA, USA). The samples were heated at 96.5° Celsius for 10 minutes, after which they were chilled on ice for 5 minutes before loading into the gel. 20 ml of the protein sample was loaded on 4-20% Criterion TGX Gels (Bio-Rad Laboratories, Hercules, CA, USA). Gels were transferred onto 0.2 mm nitrocellulose membrane (Bio-Rad Laboratories, Hercules, CA, USA) using the Trans-Blot Turbo Transfer System (Bio-Rad Laboratories, Hercules, CA, USA). After transfer, membranes were blocked for 30 minutes in 1% casein solution while gently rocking back and forth using the VWR Analog Rocker (Avantor, Radnor, PA, USA). The membranes were incubated overnight at 4° Celsius in 1:1000 PLIN-2 primary antibody (Novus Biologicals, Centennial, CO, USA), 1:500 PLIN-3 primary antibody (Proteintech, Rosemont, IL, USA), or PLIN-5 primary antibody (Novus Biologicals, Centennial, CO, USA). After incubation, the membranes were washed with PBS-T (0.05% Tween-20), three times for five minutes each wash. The membranes were then incubated for one hour at room temperature, while protected from light, in 1:1000 Goat a-rabbit IR 700 secondary antibody solution. (Bio-Rad Laboratories, Hercules, CA, USA). They were then washed with PBS-T, three times for five minutes each, and then with PBS two times for five minutes each. Membranes were imaged using a ChemiDoc XRS Imaging System (Bio-Rad Laboratories, Hercules, CA, USA).

### **Lipid Droplet Imaging**

Cell treatments: For lipid droplet imaging experiments, cells were seeded at 60,000 cells per well on a poly-L-lysine coated 12mm glass coverslip. Cells were treated with 250uM oleic acid (OA) conjugated with BSA, 10ng lipopolysaccharide (LPS), necroptotic Neuro-2A cells (nN2A), N2A plus LPS, OA plus LPS, or an untreated control. After 24 hours, media was collected for cytokine analysis and cells were fixed in 4% paraformaldehyde. Cells were stained with BODIPY and coverslips were mounted with a DAPI nuclear staining mounting media. N2A cells were cultured and split into over thirty T-75 flasks with non-filter cap lids. Once cells grew to confluence, the caps were tightened to induce hypoxia and cell death. Cells began to detach from the flasks and all apoptotic cells and media were collected, split into 8x10^6^ cells/mL aliquots and frozen for later use. Microglia were treated at a 1:10 ratio of microglia to nN2A cells during experiments.

Immunohistochemistry (IHC): Primary microglia were plated on poly-L-lysine coated glass coverslips and treated with 250uM OA for 24 hours. After PFA fixation, cells were permeabilized with 100% ice cold methanol for ten minutes at -20°C. Cells were then rinsed with 1X PBS for five minutes before blocking for 60 minutes. Primary antibody for Snx4 was added at 1:1000 and cells were incubated overnight at 4°C. Cells were washed with 1X PBS three times for five minutes and incubated with secondary antibody for 2 hours at room temperature while covered from light exposure. Cells were rinsed again in PBS, stained with BODIPY, and mounted using DAPI nuclear stain.

Imaging took place on a Nikon A1R confocal microscope. 405nm wavelength was used to acquire microglial nuclei stained with DAPI and 488nm wavelength was used to acquire neutral lipid stained with BODIPY. 560nm wavelength was used to acquire signal for Snx4 and ApoE. Color threshold analysis was used to select high intensity regions of neutral lipid, indicating the presence of lipid droplets.

**GSEA**

Gene set enrichment analysis (GSEA) was performed using the clusterProfiler R package and the MSigDB Hallmark gene set collection for mouse. Proteins were ranked by log₂ fold-change for each pairwise comparison of interest. Protein identifiers were converted to Entrez Gene IDs using the bitr function from org.Mm.eg.db. Enrichment was assessed using the GSEA function in clusterProfiler, with the minimum and maximum gene set sizes set to 5 and 500, respectively. Enrichment results were visualized using ggplot2, with normalized enrichment scores (NES), -log₁₀(p-values), and gene counts plotted across pathways.
